# Human Cytomegalovirus Antigen Presentation by HLA‐G in Infected Cells

**DOI:** 10.1111/tan.70089

**Published:** 2025-05-10

**Authors:** Mireia Altadill, Iñaki Álvarez, Michelle Ataya, Gemma Heredia, Elisenda Alari‐Pahissa, Aura Muntasell, Manuel Llano, Jonas Fuchs, Carlos Vilches, Hartmut Hengel, Anne Halenius, Miguel López‐Botet

**Affiliations:** ^1^ Department of Medicine and Life Sciences University Pompeu Fabra Barcelona Spain; ^2^ Department of Cell Biology Physiology and Immunology, Institute of Biotechnology and Biomedicine, Autonomous University of Barcelona Bellaterra Spain; ^3^ Hospital del Mar Research Institute Barcelona Spain; ^4^ Biological Sciences Department The University of Texas at El Paso El Paso USA; ^5^ Institute of Virology, Medical Center University of Freiburg Freiburg Germany; ^6^ Faculty of Medicine, University of Freiburg Freiburg Germany; ^7^ Immunogenetics and Histocompatibility Lab, Instituto de Investigación Sanitaria Puerta de Hierro ‐ Segovia de Arana Madrid Spain; ^8^ Organización Nacional de Trasplantes, Ministerio de Sanidad Madrid Spain

**Keywords:** cytomegalovirus, HLA‐E, HLA‐G, NK cell, T lymphocyte

## Abstract

HLA‐E and ‐G class Ib molecules were considered unrelated to viral antigen presentation. HLA‐E binds nonamers from the leader sequences of other HLA‐I molecules and the human cytomegalovirus (HCMV) UL40 protein, interacting with CD94/NKG2 NK cell receptors. Yet, evidence that HLA‐E may present some pathogen‐derived peptides to CD8+ T lymphocytes has been reported. By contrast, HLA‐G binds a broad spectrum of endogenous sequences but its role in antigen presentation is unknown. An experimental approach was set up to search for HCMV antigens displayed by HLA‐G in infected cells. Among the analysed peptidome, 22 sequences corresponding to 16 HCMV molecules were identified; 17 peptides were confirmed to interact in vitro with HLA‐G of which 10 displayed characteristic anchor residues. As compared to the response in short‐term (6 h) assays to immunodominant IE‐1 and pp65 antigens, none of the HLA‐G‐binding peptides stimulated cytokine production by CD8+ T cells from HCMV‐seropositive blood donors (*n* = 15). Following a 14‐day peptide stimulation of PBMC and expansion with IL‐2, CD8+ T cells specifically responding to a subset of these viral antigens were detected in some individuals, yet were not restricted by HLA‐G in functional assays. A subset of viral peptides did bind to both HLA‐G and ‐E but were not recognised by CD94/NKG2 NK cell receptors. Our results provide the first evidence that HLA‐G may display potentially immunogenic viral peptides in HCMV‐infected cells, yet do not support their ability to promote HLA‐G‐restricted CD8+ T cell responses nor to modulate NK cell functions.

## 1 Introduction

As compared to classical HLA class Ia molecules, HLA‐E and ‐G display a reduced polymorphism and were considered functionally unrelated to antigen presentation, yet studies of HLA‐E have partially challenged the paradigm. This HLA class Ib molecule, broadly expressed at low levels in different tissues [1, 2], binds conserved nonamers with Met in P2, derived from the leader sequence of other HLA‐I molecules and from the human cytomegalovirus (HCMV) UL40 protein [3, 4]. HLA‐E‐peptide complexes are recognised by inhibitory CD94/NKG2A and activating CD94/NKG2C NK cell receptors (NKR) [5, 6, 7]. We provided original evidence supporting a T‐cell receptor (TCR)‐mediated recognition of HLA‐E [8]. Subsequent studies detected a specific HLA‐E‐restricted T‐cell response to an allogeneic HLA‐I leader peptide (VMAPRTLIL), presumably primed upon infection by an HCMV strain bearing that UL40 sequence [9, 10]. Recently, a variety of human and HCMV‐derived HLA‐E‐binding peptides were identified by screening a yeast‐display library [11]. A subset of these peptide‐HLA‐E complexes engaged CD94/NKG2 NKR, promoting functional effects in NK cells, but no information was provided regarding their presentation by HCMV‐infected cells nor their ability to activate T lymphocytes. HLA‐E‐restricted T cells specific for other pathogen‐derived antigens were reported [12, 13, 14, 15] and a SARS‐CoV‐2 peptide presented by HLA‐E has been shown to be recognised by CD94/NKG2 NKR [16].

In contrast, HLA‐G displays a more restricted tissue distribution being unequivocally expressed by embryonic extravillous cytotrophoblasts (EVT) and some tumours, whereas its detection in peripheral blood mononuclear cells and other normal tissues has remained methodologically controversial [17, 18, 19]. Several HLA‐G allotypes differing in the coding region have been identified, being HLA‐G*01:01 the most frequent across different populations [20, 21]. Among different putative HLA‐G isoforms (G1‐G7) encoded by splice variants, only two display a full canonical structure expressed at the cell surface (G1) or secreted (G5) [21, 22]. An HLA‐G feature, unusually shared by other HLA‐I molecules (e.g., HLA‐B27), is its ability to form dimers by a disulphide bond between Cys residues at position 42 [23, 24, 25].

A prevailing view is that HLA‐G exerts a tolerogenic function through different mechanisms [18]. Among them, its interaction with the ILT2 (LILRB1) receptor, stronger than that of other HLA‐I molecules, has been reported to inhibit NK, T, B and myelomonocytic cells, the latter being similarly regulated by ILT4 (LILRB2) [26, 27, 28]. Mass spectrometry (MS) analyses of the HLA‐G1 peptidome in cell lines revealed a variety of endogenous sequences displaying conserved motifs [29, 30], rendering plausible that HLA‐G may also present pathogen‐derived peptides. To our knowledge, no information is available in this regard, with the exception of a report on the induction of a murine T‐cell response to peptides from the pp65 HCMV antigen in triple transgenic mice (HLA‐G, hb2m and hCD8a) [31]. Among a variety of HCMV immune evasion molecules, US2, US3, US6 and US11 downregulate HLA‐I surface expression in infected cells [32, 33, 34]. US10 was proposed to specifically target HLA‐G for proteasomal degradation [35], but has been recently reported to bind other HLA‐I molecules preventing their recruitment to the peptide loading complex as well as retaining in the ER b2m‐assembled HLA‐G and ‐C allotypes [36].

Recently, novel HCMV immunogenic epitopes were characterised by analysing the T‐cell response to HLA‐I‐bound peptides identified by MS from fibroblasts infected with viral mutants lacking immune evasion genes, which interfere with antigen presentation [37]. In the present study, we developed a similar experimental approach to explore the putative involvement of HLA‐G in HCMV antigen presentation.

## 2 Materials and Methods

### 2.1 Ethics Statement

Peripheral blood mononuclear cells (PBMC) were obtained from volunteer healthy donors. Written informed consent was obtained, and the study protocol was approved by the institutional ethics committee (Clinical Research Ethics Committee, Parc de Salut Mar CEIC 2018/7873/I).

### 2.2 Cell Lines

MSR3‐mel cells were kindly provided by Drs. Federico Garrido and Francisco Ruiz Cabello (Hospital Universitario Virgen de las Nieves, Universidad de Granada, Spain). MSR3‐mel is a melanoma cell line reported to display an epigenetic silencing of HLA‐I genes by hypermethylation (HLA‐A*01:01, A*11:01, HLA‐B*37:01, B*52:01, HLA‐C*06:02, C*12:02) [38]. MSR3‐mel cells were transfected in our lab with the pcDNA3.1‐HLA‐G1m plasmid which encodes the HLA‐G*01:01 protein containing a mutation in its signal peptide, preventing induction of cell surface expression of HLA‐E [39]. Selection and maintenance of the cell line was performed using G418 (400 μg/mL) (InvivoGen, San Diego, CA). The fibroblast cell line MRC‐5 was provided by Dr. Ana Angulo. These cell lines were grown in DMEM supplemented with 10% heat‐inactivated FBS, penicillin, streptomycin and sodium pyruvate.

The TAP2‐deficient RMA‐S murine cell line transfected either with human beta 2 microglobulin (hb2m) (herein referred to as RMA‐S/b2m), or with hb2m and HLA‐E*01:03 (RMA‐S/HLA‐E) were kindly provided in the past by Drs. M. Ulbrecht and E. Weiss (Institut für Antrophologie und Humangenetik, Ludwig‐Maximilians Universität München, Germany) [40]. RMA‐S/b2m cells were transfected with the pcDNA3.1‐HLAG1m plasmid by electroporation (GenePulser II, BioRad, 300 V and 960 μF). Selection of RMA‐S/G1m was performed using G418 (400 μg/mL) (InvivoGen, San Diego, CA). RMA‐S/G1m were cultured in complete RPMI‐1640 supplemented with G418.

### 2.3 Flow Cytometry Analysis

HLA class I expression by MSR3 and MRC5 cell lines was characterised by flow cytometry. Cells were stained with the G233 anti‐HLA‐G monoclonal antibody (mAb) (Thermo Fisher Scientific, Waltham, MA) and the W6/32 pan‐HLA‐I specific mAb (produced in our laboratory) by indirect immunofluorescence (IF) using R‐PE‐conjugated AffiniPure F(ab’)_2_ goat anti‐mouse IgG + IgM (H + L) (Jackson ImmunoResearch, West Grove, PA). Direct IF staining was carried out with: HP‐1F7 pan‐HLA‐I specific mAb (Santa Cruz Biotechnology, Santa Cruz CA) originally produced in our laboratory and conjugated to APC, TP25.99SF‐Alexa Fluor 488 anti‐HLA‐A, ‐B, ‐C, ‐E mAb (Invitrogen, Thermo Fisher, Waltham, MA) and an anti‐b2‐microglobulin‐PE mAb (Invitrogen, Thermo Fisher, Waltham, MA). Samples were analysed using a BD LSR‐II flow cytometer.

### 2.4 AB8 HCMV Characterisation and Stock Production

This work was carried out in an authorised p2‐level biohazard facility at Universitat Pompeu Fabra, in compliance with the official requirements for HCMV manipulation. AB8 HCMV (kindly provided by M. Messerle and Eva Borst, Institute for Virology, Hannover Medical School) is a recombinant virus, derived from a clinical isolate (UL1271) [41], integrated in a bacterial artificial chromosome plasmid (BACmid) that lacks the US2‐US6 gene region. Reconstituted virus was propagated in MRC‐5 cell line as described elsewhere [42]. For infecting large batches of cells, virus preparations were used as supernatant of infected MRC‐5 cells centrifuged at 1750 g for 10 min and stored at −80°C until used; viral titre was 6·10^6^ pfu/mL.

AB8 sequencing and de novo assembly was carried out as described in Supporting Information.

For data availability, raw reads have been submitted to the European Nucleotide Archive and are available under the study accession number PRJEB84051. The annotated viral genome of AB8 was submitted to NCBI (accession number: BankIt2912186 AB8, PQ867562).

### 2.5 MSR3 G1m Cell Infection

Small‐scale infections were performed in 96 w‐plates (flat bottom). Cells were seeded 24 h prior to virus addition at the indicated MOI and were incubated at 37°C. Two hours after infection, cells were washed once with phosphate buffered saline (PBS) (1×) (Gibco, Thermo Fisher, Waltham, MA) and further incubated with DMEM complete medium. For obtaining larger batches of infected cells, MSR3 G1m were grown in 20 × 175 cm^2^ flasks until ~80% confluence was reached; each flask contained approximately 2·10^7^ cells. Cells were infected with the viral supernatant prepared as described above. Two hours after infection, cells were washed with PBS and fresh complete DMEM was added. Half of the cultures were harvested at 36 h post infection (hpi) and the remaining at 48 hpi. Cell pellets were stored at −80°C.

IE‐1 staining was performed at 24 hpi in 96w‐plates (flat bottom). Cells were washed twice with PBS followed by fixation for 2 min with cold methanol (−20°C). Permeabilization was performed with 0.3% Triton X‐100 in PBS for 5 min at room temperature (RT), followed by incubation with PBS‐1% BSA for 20 min at RT. Cells were incubated with the anti‐IE1 antibody (Merck Millipore, Burlington, MA) diluted at 1:800 in PBS‐1% BSA, washed and stained with F(ab’)_2_‐goat anti‐mouse IgG (H + L) labelled with Alexa Fluor 488 (Invitrogen, ThermoFisher, Waltham, MA) diluted 1:200 in PBS‐1% BSA. After washing, samples were further incubated with a 1:5000 dilution of DAPI (1 mg/mL) and stored in darkness at 4°C until observation. Staining of IE‐1 was analysed in a Zeiss Observer fluorescence microscope (Advanced light microscopy unit, CRG) and cell count was performed with Fiji Image J (win64) software.

### 2.6 HLA Purification and Peptide Elution

Purification of HLA‐G from infected cell cultures and immunopeptidome elution was carried out as previously described for HLA‐Ia molecules [43], with some modifications. The entire process was performed at 4°C; frozen cell pellets (from about 4·10^8^ cells) were thawed in lysis buffer (LB; 20 mM Tris/HCl, pH 7.4, 150 mM NaCl, 1% IGEPAL CA‐630 (Sigma‐Aldrich)) with a cocktail of protease inhibitors (cOmplete Protease Inhibitor Cocktail, Roche Diagnostics, Basel, Switzerland) and homogenised using a potter. Lysates were incubated for 1.5 h at 4°C in a shaker and centrifuged for 10 min at 1200 g; supernatants were further ultracentrifuged for 1 h at 100,000 g. The resulting supernatants were pre‐cleared using a Tris‐blocked Sepharose column, and the flow‐through was loaded into a Sepharose column conjugated with the W6/32 IgG2a monoclonal antibody (mAb) specific for HLA class I molecules [44]. Columns were washed three times sequentially with 100 mL of NET buffer (50 mM Tris/HCl, pH 7.4, 150 mM NaCl), 10% saturated NaCl and 0.5% NP‐40; 100 mL of NET buffer with 5% saturated NaCl and 0.5% NP‐40; 300 mL of NET buffer alone and finally, with 25 mL of Tris 5 mM, pH 7.4. Peptide–HLA‐I complexes were eluted in 0.1% TFA, and 1 mL fractions were collected. Protein‐containing fractions were pooled and concentrated in a SpeedVac (Thermo Fisher Scientific, Waltham, MA); peptides were purified from HLA molecules by ultrafiltration. The obtained peptide pool was then concentrated to 100 μL, desalted in a C18 column and dried in the SpeedVac for MS analysis. The quality and purity of the preparations were controlled by SDS‐PAGE analysis of the material retained in the ultracentrifugation step, which contained the heavy chain and β2m.

### 2.7 Mass Spectrometry

Peptidome analyses were performed at the Barcelona Biomedical Research Park (PRBB) CRG/UPF Proteomics Unit, integrated in the Spanish National Research Infrastructure ICTS OmicsTech. Briefly, samples were analysed by liquid chromatography–tandem mass spectrometry (LC–MS/MS) using a 90‐min gradient in the Orbitrap Eclipse coupled to an EASY‐nLC (Thermo Fisher Scientific (Proxeon), Odense, Denmark) in data‐dependent acquisition (DDA) mode. Each sample was injected twice, in one injection 30% of the sample was analysed with a method where fragment ion spectra were produced via high‐energy collision dissociation (HCD) and another 30% was analysed with a method where fragment ion spectra were produced via electron‐transfer/higher‐energy collision dissociation (EThcD). Digested bovine serum albumin (New England Biolabs cat #P8108S) was analysed between each sample to avoid sample carryover and to assure stability of the instrument, and QCloud [45] has been used to control instrument longitudinal performance during the project.

The results were searched against both a SwissProt Human database (October 2019) and a combination of the Swiss Prot Human database with the CMV proteome (UP000122942) plus the more common contaminants using the MaxQuant software (v 1.6.2.6) with an unspecific search and limiting the number of residues from 8 to 12. Peptides were filtered based on a false discovery rate (FDR) lower than 5%. To check whether the identified peptides fit with the described peptide motifs for HLA‐G, they were aligned using the Seq2Logo v2.0 software [46] with the Kullback–Leibler logo type and the default settings.

### 2.8 Peptide Synthesis

HCMV peptides without end modifications were synthesised in the Peptide Synthesis Facility (Universitat Pompeu Fabra, UPF). Purity of 98% was assessed by HPLC. Lyophilized peptides were then resuspended with H_2_O at 10 mM, aliquoted and stored at −20°C until use.

### 2.9 In Silico Prediction of Peptide‐HLA‐I Interactions and In Vitro Binding Assays

Peptide‐HLA‐I interactions were analysed using the NetMHCpan‐4.1 server (https://services.healthtech.dtu.dk/services/NetMHCpan‐4.1/) [47]. Thresholds for strong and weak binders were set to % rank 0.5 and 2, respectively.

RMA‐S/G1m and ‐/HLA‐E transfectants were used to check binding of the synthesised peptides. Cells were incubated 18–20 h at 26°C in complete RPMI 1640, washed three times in PBS and resuspended in serum‐free RPMI 1640 at a concentration of 2·10^6^ cells/mL. Cells were incubated for 1 h at 26°C in the absence or presence of peptides at different concentrations ranging from 0.01 to 300 μM in a 96 w‐plate (U bottom) and further incubated at 37°C for 4 h. Indirect immunofluorescence staining with the anti‐HLA‐G antibody G233 (1 μg/mL) (Thermo Fisher Scientific, Waltham, MA) was performed. R‐PE‐conjugated AffiniPure F(ab’)_2_ Fragment goat anti‐mouse IgG + IgM (H + L) (Jackson ImmunoResearch, West Grove, PA) was used as a secondary antibody. Cells were fixed with the BD Cytofix/Cytoperm fixation/permeabilization kit (BD Biosciences) and analysed in a BD LSR II Flow Cytometer.

### 2.10 T‐Cell Cytokine Production Assays

To analyse cytokine production by CD8+ T cells upon stimulation with HCMV‐derived peptides, PBMC were obtained by Ficoll density gradient centrifugation of blood from 15 healthy donors (9 men and 6 women, 2 reporting previous pregnancies) and cryopreserved until used. Cells were thawed and kept o/n in a polypropylene tube with complete RPMI supplemented with 50 UI/mL of recombinant human interleukin‐2 (rhIL‐2; Proleukin, Chiron, Emeryville, CA). Samples were incubated either alone with peptides (100 μM) or in the presence of RMA‐S/G1m or ‐/b2m transfectants previously loaded o/n at 26°C with peptides (100 μM). When indicated, blocking of the ILT2 (LILRB1) inhibitory receptor was achieved with the anti‐ILT2 (clone HP‐F1, IgG1) mAb employed as concentrated hybridoma supernatant [39, 48]. Cells were pre‐incubated for 30 min at RT with HP‐F1, maintained during the whole assay. As positive control, PBMC were stimulated with 1 μg/mL of mixed peptide libraries from CMV IE‐1 and pp65 antigens (PepTivator, Miltenyi Biotec). The assay was performed for 6 h in a 96 w‐plate (U bottom) with a final volume of 125 μL/w in the presence of CD49d (1 μg/mL) (BD Biosciences, San Jose, CA). Brefeldin A (Sigma‐Aldrich, Saint Louis, MO) was added during the last 5‐h at a final concentration of 10 μg/mL and a final volume of 250 μL [49]. Subsequently, cells were stained with anti‐CD3‐PerCP (clone SK7, BD), ‐CD56‐BV605 (clone NCAM16.2, BD Horizon), ‐CD4‐APC (clone RPA‐T4, BD Pharmingen) and ‐CD8‐V500 (clone RPA‐T8, BD Horizon). Cells were fixed using the BD Cytofix/Cytoperm fixation/permeabilization kit (BD Biosciences, San Jose, CA) and stained with anti‐TNFα (Infliximab) directly labelled with CF‐Blue (Immunostep Salamanca, Spain) and anti‐IFNγ‐PE (clone B27, BD Biosciences, San Jose, CA) antibodies. Cell viability was assessed with LiveDead Fixable Near‐IR dead cell stain kit (Invitrogen, Thermo Fisher, Waltham, MA), and samples were analysed on a BD LSR Fortessa flow cytometer.

### 2.11 T‐Cell Stimulation and Expansion

Based on a previous report [37], PBMC were cultured for 14 days in the presence of 100 μM HCMV peptides with or without RMA‐S/G1m, ‐/b2m or ‐/HLA‐E irradiated at 40 Gy. Library pools of HCMV IE‐1 and pp65 antigens (PepTivator, Miltenyi Biotec) were used as positive controls of specific activation and expansion. Cultures were performed in 48 w‐plates at a cell density of 5·10^5^ PBMC/w; RMA‐S transfectants were included at a 10:1 ratio (PBMC:RMA‐S). Cultures were supplemented with rhIL‐2 (200 UI/mL) at days 1, 3, 8 and 10. When indicated, re‐stimulation with fresh peptide (100 μM) and irradiated RMA‐S transfectants was carried out on Day 6. Functional assays were performed at days 0, 6 and 14 following the protocol indicated above.

### 2.12 NK Cell Expansions and Degranulation Assays

As described, PBMC were co‐cultured in the presence of the irradiated RPMI‐8866 cell line [50] at a ratio 3:1 (3 × 10^6^ PBMCs and 1 × 10^6^ 8866 cells per well in a 12 well‐plate) in complete RPMI 1640 GlutaMax (Thermo Fisher Scientific) for 12 days. Cultures were split and fed every 3–4 days, adding IL‐2 (200 U/mL) from Day 7. The phenotype of expanded NK cells was checked at days 6, 10 and 12 by analysing CD3, CD56, NKG2C and NKG2A expression (BD LSR Fortessa). To assess NK cell degranulation, PBMC or NK cells were co‐cultured with RMA‐S transfectants previously loaded with peptides (100 μM) o/n at 26°C in a 96 w‐plate (U bottom) (100 μL/w); samples were pre‐treated for 30 min at RT with anti‐ILT2 mAb (clone HP‐F1) prior to stimulation. Co‐cultures were performed at a 4:1 E/T ratio in a 96w‐plate (U bottom) at a final volume of 200 μL/w. Anti‐CD107a‐APC (clone H4A3, BD FastImmune, San Jose, CA) and 5 μg/mL monensin were present in the co‐culture during the whole assay. After incubation for 4 h at 37°C, cells were stained with anti‐CD3‐PerCP (clone SK7, BD), ‐CD56‐BV605 (clone NCAM16.2, BD Biosciences, San Jose, CA), ‐NKG2A‐Pacific Blue (clone Z199 provided by Dr. A. Moretta), and ‐NKG2C‐PE (clone FAB138P, R&D systems, Bio‐Techne, Minneapolis, MN) and analysed in a BD LSR Fortessa flow cytometer.

## 3 Results

### 3.1 Identification of HCMV Peptides Presented by HLA‐G: Experimental Design

**FIGURE 1:**
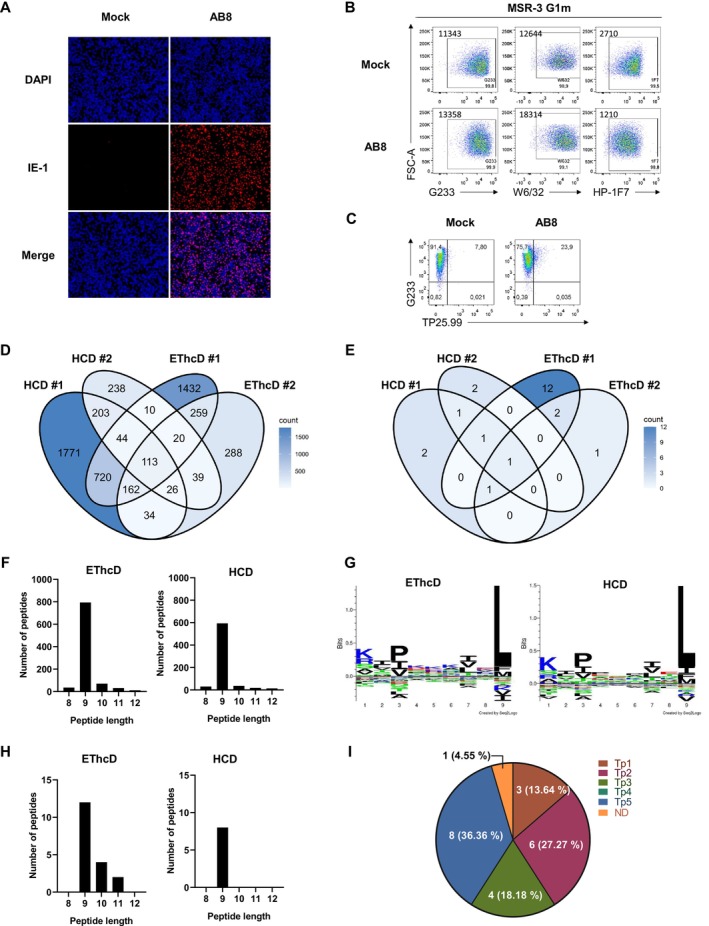
Identification of HCMV peptides eluted from MSR3 G1m infected cells. (A) Representative IE‐1 staining (red) HCMV infected cells (MOI 5) at 24 hpi. (B) Flow cytometry analysis of mock and HCMV‐infected MSR3 G1m cells stained by indirect immunofluorescence with G233 HLA‐G‐specific or W6/32 and by direct immunofluorescence HP‐1F7 anti‐HLA‐I mAbs. Populations are gated according to the negative control (not shown). Percentage of positive cells and MFI of the whole population are shown. (C) G233 and TP25.99SF combined staining of infected (AB8) and non‐infected (Mock) MSR3 G1m cells at 48 hpi. Populations are gated according to the negative control (not shown). (D) Overlap analysis of total number of peptides identified by HCD or EThcD breaking methods from two different infection batches at 36–48 hpi. (E) Overlap analysis of HCMV‐peptides identified by HCD or EThcD from two different infection batches at 36–48 hpi. (F) Histograms from elution #2 showing the length of peptides identified by both methods. (G) Seq2Logo of peptides eluted in batch #2 comparing both methods. (H) Distribution according to their length of HCMV peptides, identified in two elutions obtained by EThcD and HCD. (I) Distribution of the corresponding viral proteins categorised according to their temporal expression along the lytic cycle according to Weekes et al. [51].

To explore HCMV antigen presentation by HLA‐G, we anticipated the need of a cell line permissive for HCMV infection, selectively expressing the class Ib molecule to allow its purification by affinity chromatography with the available W6/32 mAb specific for HLA‐I molecules. On that basis we selected MSR3‐mel, a melanoma‐derived cell line reported to display an epigenetic silencing of HLA‐I genes by hypermethylation, also preventing the presence of HLA‐I leader sequences binding to HLA‐E [38]. MSR3‐mel cells were transfected with pcDNA3.1‐G1m, a plasmid which encodes HLA‐G with a non‐synonymous mutation in the leader sequence, avoiding its loading onto HLA‐E [39]. MSR3 appeared refractory to infection by commonly available HCMV strains (i.e., AD169, Towne, TB40/E) (not shown) but was permissive to a strain derived from a clinical isolate (UL1271) (Figure 1A) available as a recombinant BACmid clone (AB8) [41], in which US2, US3 and US6 genes are deleted, thus reducing HLA‐I downregulation in infected cells as previously shown for the AD169 strain BAC (HB5) [52]. Of note, AB8 maintains other immune evasins targeting HLA‐I (i.e., US10, US11) (Figure S1), and US10 has been reported to interfere with HLA‐G expression [35, 36].

Characterisation by flow cytometry analysis of MSR3 G1m showed a comparable staining with the HLA‐G‐specific G233 and two pan‐HLA class I‐reactive mAbs, HP‐1F7 and W6/32 (Figure 1B). To directly assess the putative re‐expression of MSR3 endogenous HLA‐I molecules in MSR3 G1m cells prior and after HCMV‐infection, two‐colour flow cytometry analysis was carried out combining HP‐1F7 and TP25.99, a mAb reported to specifically react with HLA‐A, ‐B, ‐C and ‐E molecules but not HLA‐G [53]. Detection of TP25.99+ cells was marginal in MSR3 G1m cells as compared to their dominant HLA‐G1 expression (Figure 1C); by contrast TP25.99 and HP‐1F7 staining was similar in control MRC5 fibroblasts, as expected (Figure S2). On the other hand, HLA‐G was confirmed to be assembled with b2m in MSR3 G1m cells (Figure S2). Of note, a minimal effect of infection on HLA‐G surface levels was perceived in MSR3 G1m cells, brightly stained by indirect IF with either G233, and anti‐HLA‐I W6/32 (Figure S2). However, a reduction of the lower direct IF staining of HLA‐I and b2m was noticed in infected cells (Figure S2), suggesting an effect of US10.

### 3.2 Detection of HLA‐I‐Bound Peptides From HCMV‐Infected MSR3 G1m Cells

MSR3 G1m cells were infected with AB8 HCMV, and HLA‐I was purified by affinity chromatography. Two different infection batches (#1, #2) were sequentially generated, each containing a mixture of cells harvested at 36 and 48 h post‐infection (hpi). Conditions for HLA‐I purification, peptide elution and MS analysis were set up, combining two different peptide breaking methods: electron‐transfer/EThcD and HCD, which yielded complementary data.

In batch #1 > 4700 unique peptides were detected, including 20 HCMV sequences. In batch #2, out of 1436 peptides 9 were from HCMV, 6 coinciding with those in batch #1 (Figure 1D,E). The infection rate attained with AB8 HCMV after infection of 10^8^ cells was reduced to around 40%, indicating that a substantial proportion of human peptides were from non‐infected cells, and the ratio of viral versus endogenous peptides from infected cells could not be estimated. Though the experimental conditions might be further refined, characterisation of the identified viral peptides was prioritised.

Most eluted peptides were nonamers, though some 8‐, 10‐, 11‐ and 12‐mers were also detected (Figure 1F). Analysis of residue distribution in total 9‐mers revealed a pattern coincident with sequence motifs previously reported for HLA‐G (Figure 1G) [30]. Most HCMV peptides were identified in batch #1 using EThcD and included 9‐ (*n* = 16), 10‐ (*n* = 4) and 11‐mers (*n* = 2) (Figure 1H), corresponding to 15 different viral proteins, displayed along the whole lytic cycle according to Stern‐Ginossar et al. [54] and Weekes et al. [51] (Figure 1I, Table S3). Identification of peptides from late proteins and detection by immunofluorescence of gB in AB8 infected MSR3 G1m cells (data not shown) confirmed that the virus was able to complete the lytic cycle.

Endogenous HLA‐G‐binding nonamers were reported to contain conserved residues in P3 and P9, corresponding to anchor positions [30]. A comparison of HCMV peptide sequences (*n* = 22) (Table 1) revealed that 13 contained as well proline (P) or valine (V) in P3; and 11 showed leucine (L) at P9, also found at the C‐terminal position (Ω) of 10‐mers and 11‐mers; 10 peptides displayed both anchor residues at P3 and P9/P10/P11. Moreover, nonamers binding to HLA‐G were also reported to contain auxiliary anchor residues at P1, namely lysine (K) and arginine (R) [29], being detected in 8 HCMV peptides. In silico prediction of interactions with HLA‐G was carried out through the NetMHCpan4.1 server [47]. According to their individual binding rank calculated by this approach, peptides were categorised as strong (UL69 (HQ9L), US11 (SM9L), UL31 (VQ9L), UL148 (VS9L), UL69 (RL9L), UL13 (RE9F), UL54 (KI9F), US33A (RF11L)) or weak binders (UL38 (VRP9L), UL111A (VM9L), UL54 (HL9L), UL54 (QI9F), UL36 (RT10L)), whereas the remaining nine were not predicted to interact with HLA‐G (Table 1). Interestingly, SM9L derived from the US11 immune evasin was previously reported to bind as well to HLA‐A*02 [55].

**TABLE 1:** HCMV peptides identified from infected MSR3 G1m cells.

Peptide	Sequence											NetMHCpan4.1	Binding to RMA‐S/G1m			Specific T‐cell responses
	1	2	3	4	5	6	7	8	9	10	11					
VRP9L	V	R	P	T	R	Q	L	V	L			0.5699		^a^		
HQ9L	H	Q	P	R	G	R	I	L	L			0.0717		^a^		
HL9L	H	L	V	P	S	G	N	V	L			0.9338		^a^		
KR9L	K	R	A	M	Y	S	V	E	L			2.5473		^a^		X
SM9L	S	M	P	E	L	S	L	T	L			0.01		^a^		
RF11L	R	F	P	E	R	A	G	Y	E	K	L	0.0715		^a^		
QI9F	Q	I	V	P	R	G	V	M	F			0.52		^b^		X
VRS9L	V	R	S	R	D	S	L	L	L			2.8248		^b^		X
RI11L	R	I	V	E	P	L	E	S	G	R	L	4.0386		^b^		X
VQ9L	V	Q	P	R	Q	T	V	E	L			0.0487		^b^		X
VS9L	V	S	P	G	K	E	V	T	L			0.0491		^b^		
SG10L	S	G	V	R	R	P	F	T	E	L		3.7952		^b^		X
TL10L	T	L	K	G	L	R	K	L	I	L		9.3978		^b^		X
KI9F	K	I	P	L	R	R	V	I	F			0.0971		^b^		X
RT10L	R	T	G	S	L	H	H	F	E	L		1.598		^b^		X
VM9L	V	M	V	S	S	S	L	V	L			1.3497		^b^		X
RL9L	R	L	A	P	Y	P	A	D	L			0.3077		^b^		X
SP10L	S	P	S	R	D	R	F	V	Q	L		4.3484				X
SE9V	S	E	T	T	V	H	V	V	V			22.1101				X
VR9K	V	R	L	S	D	L	R	L	K			43.6364				X
RP9L	R	P	R	L	T	L	H	D	L			5.3686				X
RE9F	R	E	P	P	H	R	A	L	F			0.1598				

*Note:* Orange: HLA‐G anchor residues. Green: HLA‐G preferred residues. Dark blue: Strong binder to HLA‐G according to NetMHCpan4.1 (< 0.5% rank). Light blue: Weak binder to HLA‐G according to NetMHCpan4.1 (0.5% < *x* < 2% rank).

^a^ Strong in vitro binding to HLA‐G.

^b^ Weak in vitro binding to HLA‐G.

### 3.3 In Vitro Analysis of HCMV Peptide Binding to HLA‐G

**FIGURE 2:**
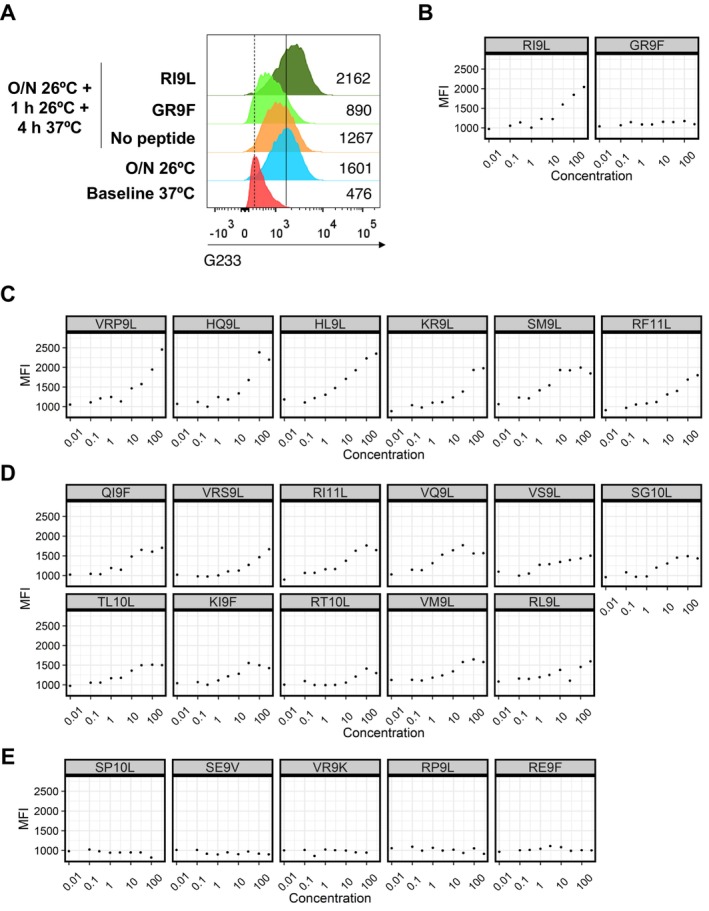
Analysis of HCMV peptides binding to RMA‐S/G1m cells. (A) Flow cytometry analysis of RMA‐S/G1m stained by G233 anti‐HLA‐G mAb pre‐ and post‐ in vitro loading with the indicated synthetic peptides. MFI values are inserted besides each histogram. Dotted line marks the peak of the baseline condition at 37°C, and the solid line is aligned with the peak after o/n incubation at 26°C, both without peptide. (B) Binding curve of positive (RI9L) and negative (GR9F) control peptides. Flow cytometry analysis was performed after indirect immunofluorescence staining with the G233 mAb. (C–E) Binding curves of peptides showing strong (C), weak (D) and no‐interaction (E).

To directly assess the ability of viral peptides to bind HLA‐G, a conventional method was employed expressing HLA‐G in the TAP‐2 deficient RMA‐S murine cell line, previously transfected with human b2m (RMA‐S/b2m). RMA‐S/G1m cells were preincubated at 26°C with different concentrations (0.01–300 μM) of synthetic peptides corresponding to the identified viral sequences. Peptide stabilisation of HLA‐G surface expression at 37°C was assessed by flow cytometry with the G233 mAb. The GRLTKHTKF (GR9F, from rat ribosomal protein L36_36‐44_) and RIIPRHLQL (RI9L, from histone H2A_77‐85_) peptides were, respectively, included as negative and positive controls (Figure 2A,B) [56]. Based on the ratio between maximum and baseline MFI, viral peptides were arbitrarily categorised as strong (> 1.8) or weak (1.3 < *x* < 1.8) binders (Figure 2C,D), while five failed to stabilise HLA‐G expression (Figure 2E), consistent with their lack of both anchor residues.

Substantial differences were noticed between in vitro RMA‐S/G1m‐binding peptides and NetMHCpan4.1‐based predictions (Table 1). In fact, among eight peptides categorised in silico as strong binders, four showed a weak in vitro interaction (UL31 (VQ9L), UL148 (VS9L), UL54 (KI9F), UL69 (RL9L)) and one (UL13 (RE9F)) did not detectably bind to RMA‐S/G1m cells. On the other hand, among five weak binders predicted by NetMHCpan4.1 (UL38 (VRP9L), UL111A (VM9L), UL54 (HL9L), UL54 (QI9F), UL36 (RT10L)), 2 (UL38 (VRP9L) and UL54 (HL9L)) displayed a strong in vitro interaction with RMA‐S/G1m. Finally, out of nine peptides not predicted to bind HLA‐G, five stabilised its expression in RMA‐S/G1m cells (UL78 (KR9L), UL13 (VRS9L), UL148 (RI11L), UL148 (SG10L) and US26 (TL10L)). These discordances between in vitro observations and in silico predictions raised some caution.

The possibility that residual endogenous HLA‐Ia molecules in MSR3 G1m cells, despite their marginal expression (Figure 1C) might have been co‐purified with HLA‐G was considered. The putative interaction of viral peptides with endogenous MSR3 HLA‐I molecules was explored using NetMHCpan4.1, and five nonamers interacting with HLA‐G were predicted to preferentially bind to some MSR3 HLA‐I molecules. On the other hand, six peptides, including three not binding to HLA‐G in vitro, were not assignable to any other HLA‐I molecules, thus leaving their origin uncertain (Table S4).

### 3.4 Analysis of the T‐Cell Response to HCMV Peptides

**FIGURE 3:**
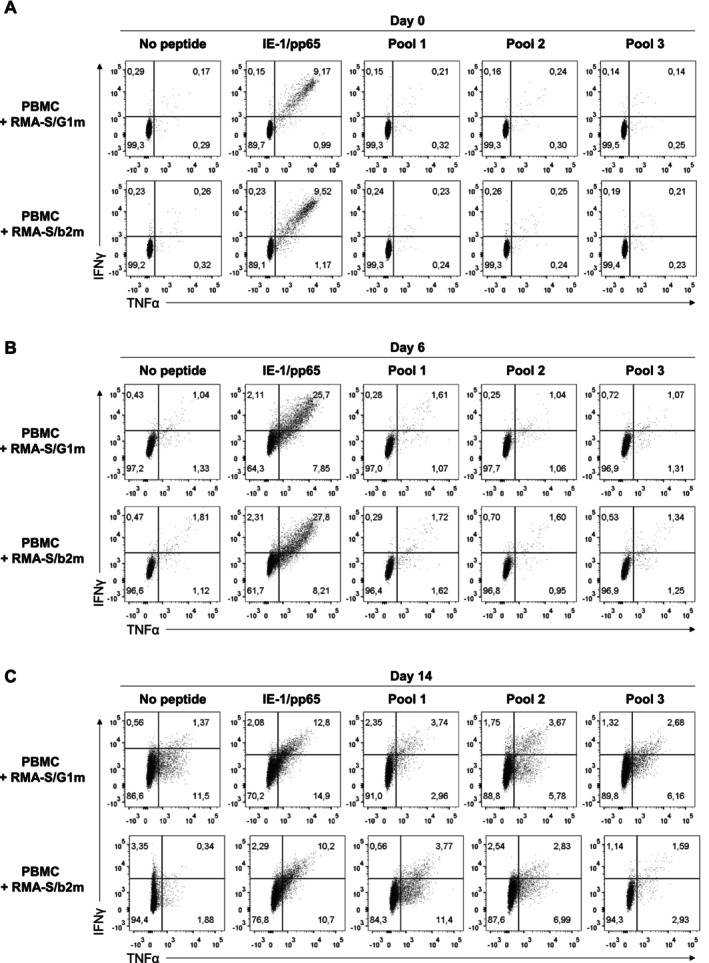
Cytokine production by CD8+ T cells in response to stimulation and expansion with HCMV peptide pools. PBMC were co‐cultured with irradiated RMA‐S/G1m or RMA‐S/b2m cells, pre‐loaded with HCMV peptide pools (Figure 2C–E) in the presence of rhIL‐2 (see Section 2). Cells were re‐stimulated at Day 6 with fresh peptide‐loaded RMA‐S/G1m or RMA‐S/b2m. PBMC incubated with the IE‐1/pp65 peptide pool was included as a positive control. Cells were harvested and analysed for TNFα and IFNγ secretion at days 0 (A), 6 (B) and 14 (C) after co‐culture.

To explore the presence of circulating T cells capable of responding to the identified viral peptides, PBMC from HCMV+ blood donors were stimulated with RMA‐S/G1m cells loaded with three peptide pools, grouped according to their in vitro binding strength to HLA‐G. T‐cell response was analysed by intracellular staining of IFNγ and TNFα after 6 h of co‐culture. No T‐cell activation was detected, as compared to the control response of PBMC incubated with peptide libraries from IE‐1 and pp65 immunodominant HCMV antigens (Figure 3A).

In a recent report [37], specific T‐cell activation by HCMV antigens, undetectable in short‐term assays, could be evidenced following a prolonged peptide stimulation and expansion in culture. A similar strategy was applied, culturing PBMC from HCMV+ donors for 14 days in the presence of irradiated RMA‐S/G1m or RMA‐S/b2m loaded with HCMV peptide pools and rhIL‐2; peptides were not washed out and cultures were re‐stimulated by Day 6 under the same conditions. The CD8+ T‐cell response to IE‐1 and pp65 HCMV antigens co‐cultured in the presence of RMA‐S/G1m or RMA‐S/b2m was tested in parallel. As expected, increased proportions of specific cytokine‐producing cells to these immunodominant antigens were detected, yet regardless of the presence of RMA‐S/G1m cells (Figure 3B,C).

Despite a peptide‐independent activation background observed by Day 14 in some cultures with RMA‐S cells, a specific response of CD8+ T cells to peptide pools 1 and/or 2 was identified in some donors (Figure 3). However, background activation detected also in the presence of RMA‐S/b2m questioned the role of RMA‐S/G1m, indicating that viral antigens might be presented either by HLA‐G and/or by other HLA‐I molecules present in PBMC.

**FIGURE 4:**
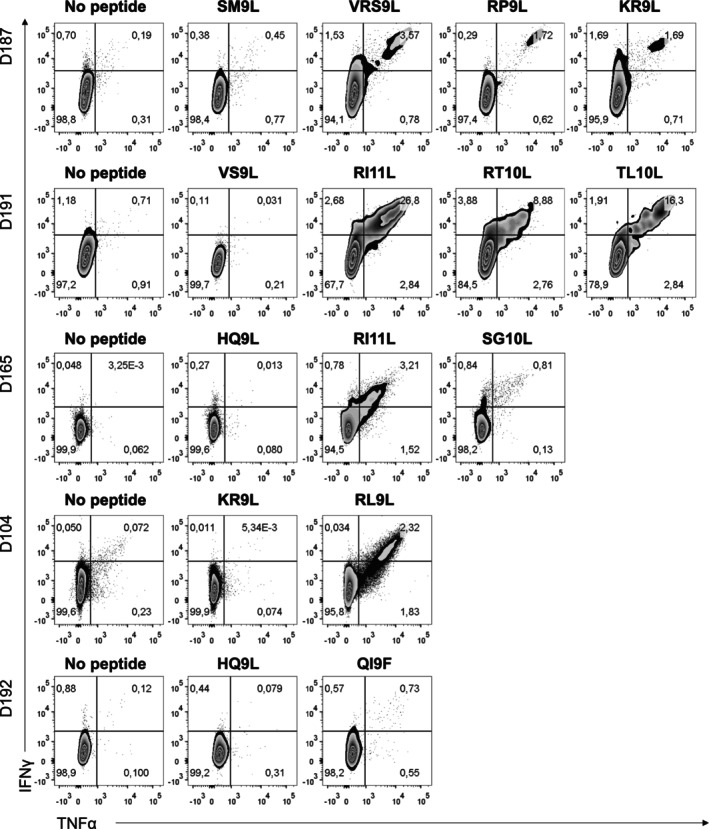
CD8+ T cell response of HCMV+ blood donors to stimulation and expansion with individual HCMV peptides. Representative zebra plots of cytokine production by CD8+ T cells of five different donors in response to HCMV peptides. IE‐1/pp65 peptides were used as a positive control of specific‐T cell expansion (not shown). For each donor, lack of response in the presence of a non‐stimulating peptide (D187‐US11 (SM9L), D191‐UL148 (VS9L), D165‐UL69 (HQ9L), D104‐UL78 (KR9L), and D192‐UL69 (HQ9L)) is shown.

To address this issue, PBMC from selected HLA‐I genotyped HCMV+ donors, 14 homozygous for G*01:01 and one G*01:01/*01:03, were stimulated with individual viral peptides in the absence of RMA‐S for 2 weeks in the presence of rhIL‐2; cytokine production assays in response to a 6 h peptide stimulation were performed at days 0, 6 and 14. As described above, only activation with the IE‐1/pp65 peptide pool was detectable at Day 0, with variable proportions of responding TNFα+ IFNγ+ T cells in different individuals (data not shown). Remarkably, T cells specifically producing cytokines in response to different viral peptides were detected by Day 14 in some donors (Figures 4 and S5).

**FIGURE 5:**
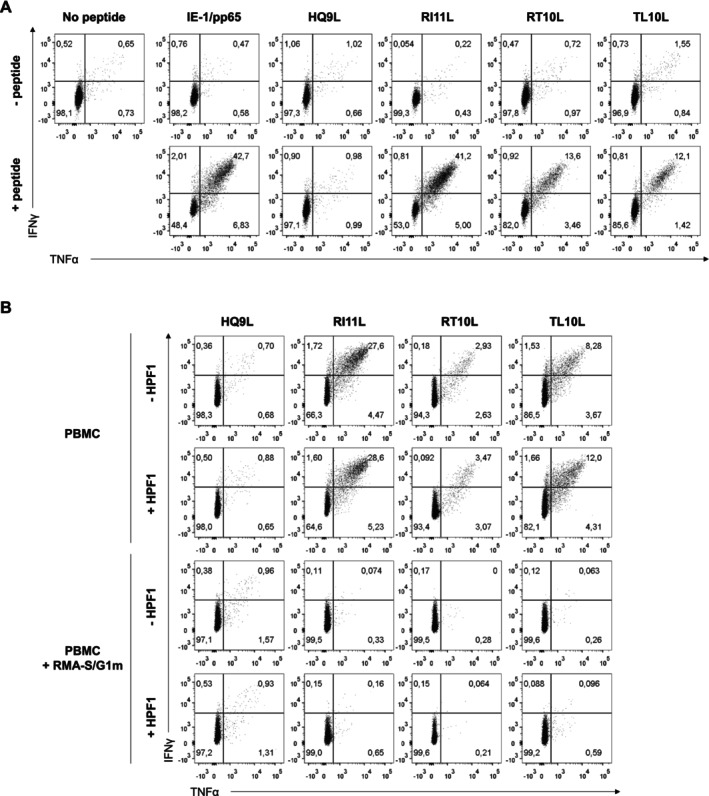
Expanded HCMV peptide‐specific CD8+ T cells do not respond to re‐stimulation by RMA‐S/G1m‐bound peptides. (A) Analysis of cytokine production by CD8+ T cells following peptide stimulation and expansion was carried out in the presence or absence of the peptide during the functional assay. Negative (no peptide) and positive (IE‐1/pp65) controls, as well as CD8+ T cell responses to activating (UL148 (RI11L), UL36 (RT10L), US26 (TL10L)) and non‐activating (UL69 (HQ9L)) peptides are shown. (B) PBMC from a donor showing T cell expansions following stimulation by HCMV peptides (UL148 (RI11L), UL36 (RT10L), and US26 (TL10L)) were re‐challenged with the same antigens (100 μM), either soluble or pre‐loaded onto RMA‐S/G1m cells and washed; the response to a non‐activating control peptide (UL69 (HQ9L)) is shown. The assay was carried out in the absence or presence of anti‐ILT2 mAb (HP‐F1). A representative result of three experiments is shown.

Peptide re‐stimulation during the last 6 h was required to detect T‐cell activation in response to either IE‐1/pp65 or the identified viral antigens (Figure 5A). To assess whether ILT2 interaction with HLA‐I molecules might interfere with the response, re‐stimulation assays were performed in the presence of the blocking HP‐F1 mAb, which minimally changed the proportions of cytokine‐producing cells. On the other hand, T‐cell activation in response to HCMV peptides was not blocked by anti HLA‐G mAbs (i.e., G233, 87G; data not shown); a caveat was the lack of positive control, given the unprecedented identification of HLA‐G restricted T cells. Importantly, activation was not observed when cells were re‐challenged with peptides displayed by RMA‐S/G1m, regardless of the presence of anti ILT2 mAb, excluding that their unresponsiveness might result from HLA‐G engagement of the inhibitory receptor (Figure 5B). Of note, RMA‐S/G1m cells bound to viral peptides did not inhibit the response of samples of different donors to IE‐1/pp65 antigens (data not shown).

In summary, the response to individual HCMV peptides and their magnitude varied in 15 HCMV+ donors. In every case, a variable increase in the numbers of TNFα+ IFNγ+ producing cells was observed after in vitro expansion in response to IE‐1/pp65 peptide pools. By contrast, specific T‐cell responses to some viral peptides were only detected in 10 individuals ranging between 0.7% and 4%, with a single case reaching 22% cytokine‐producing CD8+ cells at day 14. Of note, some peptides (UL78 (KR9L), UL54 (QI9F), UL13 (VRS9L), UL148 (RI11L), US26 (TL10L), UL36 (RT10L), UL103 (VR9K) and UL53 (RP9L)) triggered a detectable T‐cell response in more than one donor (Tables 1 and S6).

Altogether these observations questioned the role of HLA‐G in peptide stimulation, rather pointing to a possible involvement of endogenous HLA‐I molecules. The putative interaction of activating peptides with HLA‐I allotypes from every donor was assessed in silico by NetMHCpan‐4.1; a representative analysis is shown in Table S7. Some peptides categorised as strong/weak binders for HLA‐G were predicted to interact as well with other HLA‐I molecules. Of note, coincidences were more frequent for HLA‐C than for HLA‐A or ‐B alleles and, remarkably, 14 out of the 22 HCMV peptides were also predicted to interact with HLA‐E.

Similar analyses were carried out for all responding donors considering their individual HLA‐I genotype (data not shown). Among three individuals responding to UL78 (KR9L), binding predictions pointed to C*07:01 for two and B*14:01 for the third; actually, this peptide was already described to be presented by C*07:01 by Lübke et al. [37]. Of note, among strong HLA‐G‐binding peptides, this was the only one which triggered a T‐cell response. Activation by UL54 (QI9F), UL31 (VQ9L), UL111A (VM9L) and UL69 (RL9L) preferentially pointed to G*01:01, according to NetMHCpan4.1, despite that these peptides might also bind with a lower score to other HLA‐I molecules. The coincident responses to UL36 (RT10L) detected in three donors (D179, D191, D202) could only be related with its weak in vitro binding to G*01:01, as this peptide was not predicted to interact with any other HLA‐I molecules. T‐cell activation elicited by other UL36‐derived epitopes has been recently described [37, 57] raising interest in the immunogenic potential of this ORF. Similarly to UL36 (RT10L), in silico analysis did not explain the responses to US26 (TL10L) and UL103 (VR9K), detected in three and two donors respectively, as these peptides were not predicted to bind any of their HLA‐Ia molecules (Table S6).

In summary, 13 epitopes identified in our study were not previously described and are not listed in the Immune Epitope Database and Analysis Resource (IEDB); 9 did bind to HLA‐G in vitro, despite that NetMHCpan‐4.1 prediction was not preferentially assigned to G*01:01. On the other hand, five were derived from 4 immunogenic antigens previously reported by Sylwester et al. [49], including UL78 (KR9L) and UL32 (SP10L) described by Lübke et al. [37]. Moreover, a nonamer (UL13 (VRS9L)) was part of an immunogenic 15‐mer peptide described by Dhanwani et al. [58] (Table S6).

Despite that our data support the immunogenicity of some antigens identified by their ability to bind to HLA‐G, limitations of in silico analysis together with the relative promiscuity of peptide‐HLA‐I interactions and, particularly, the inability to boost the responses with peptide‐loaded RMA‐S/G1m cells questioned an involvement of the class Ib molecule to promote a specific CD8+ T cell mediated response to these antigens.

### 3.5 Analysis of HCMV‐Peptides Binding to HLA‐E

**FIGURE 6:**
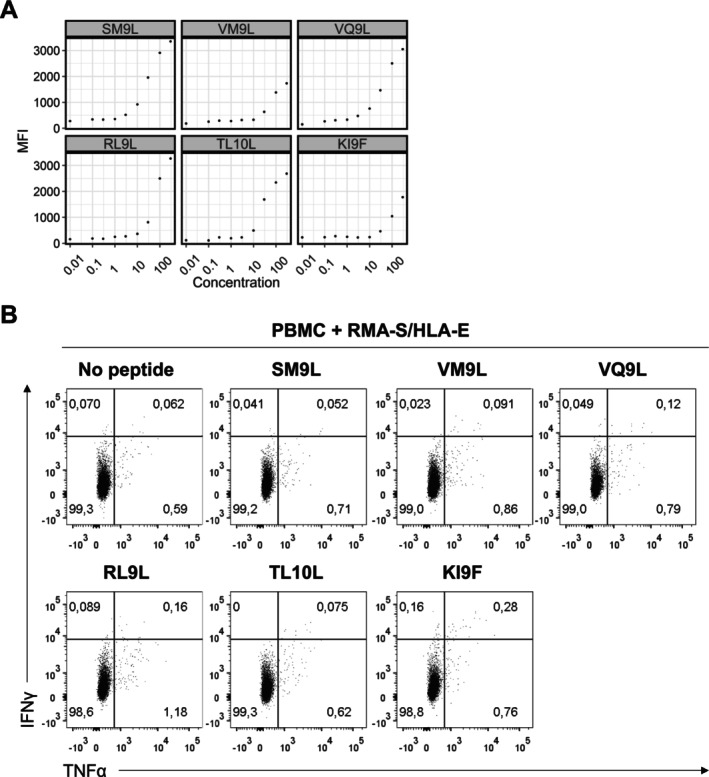
HLA‐G1‐bound HCMV peptides interacting with HLA‐E do not stimulate CD8+ T cells. (A) Binding profiles of HCMV peptides interacting with RMA‐S/HLA‐E cells at concentrations ranging from 0.01 to 300 μM. Flow cytometry analysis was performed with the HLA‐E‐specific 3D12‐PE mAb. (B) PBMC were co‐cultured for 14 days in the presence of IL‐2 with irradiated RMA‐S/HLA‐E cells previously loaded with HCMV peptides shown in panel A (100 μM) (see Methods). At day 14, cells were stimulated for 6 h with RMA‐S/HLA‐E previously loaded with HCMV peptides and washed; cytokine production was analysed. A representative result of three experiments is shown.

Since some HCMV peptides eluted from MSR3 G1m cells were predicted to bind HLA‐E, their interaction with RMA‐S/HLA‐E cells was tested in vitro, again with discordant results between both assays. In contrast with in silico predictions which categorised 11 of the 22 HCMV peptides as HLA‐E binders, only 5 of them (US11 (SM9L), UL111A (VM9L), UL31 (VQ9L), UL69 (RL9L) and UL54 (KI9F)) and another one considered no binder US26 (TL10L) were confirmed to interact in vitro with both HLA‐E and HLA‐G (Figure 6A, Table S7). T‐cell responses to UL69 (RL9L) and US26 (TL10L) were respectively observed in one and two donors (data not shown). However, specific T‐cell activation was undetectable in PBMC from three individuals expanded and re‐stimulated with RMA‐S/HLA‐E loaded with these HCMV peptides (Figure 6B).

### 3.6 HLA‐G and HLA‐E Interaction With HLA‐I Leader Sequence Peptides: Analysis of the Specific T Cell Response

As described in the previous section, among HLA‐G binding viral peptides predicted in silico to interact with HLA‐E, only some were confirmed in vitro to bind both HLA‐Ib molecules. Interestingly, in silico analysis predicted a reciprocal interaction of HLA‐G with HLA‐E bound nonamers, derived from HLA‐I leader sequences and the HCMV UL40 protein (Table 2).

**TABLE 2:** In silico interaction prediction and in vitro binding of HLA‐I‐derived signal peptides to HLA‐G and HLA‐E.

Sequence	NetMHCpan4.1 G*01:01	Binding to RMA‐S/G1m	NetMHCpan4.1 E*01:01	Binding to RMA‐S/HLA‐E
VMAPRTLIL	0.0683	^a^	0.0041	^a^
VMAPRTLFL	0.0338	^a^	0.0043	^a^
VMAPRTVLL	0.0215	^a^	0.0035	^a^
VMAPRTLVL	0.0418	^a^	0.0034	^a^
VMAPRTLLL	0.0278	^a^	0.0031	^a^
VTAPRTLLL	0.0612	^a^	0.0088	^a^

*Note:* Dark blue: Strong binder according to NetMHCpan4.1 (< 0.5% rank).

^a^ Strong in vitro binding to HLA‐G.

**FIGURE 7:**
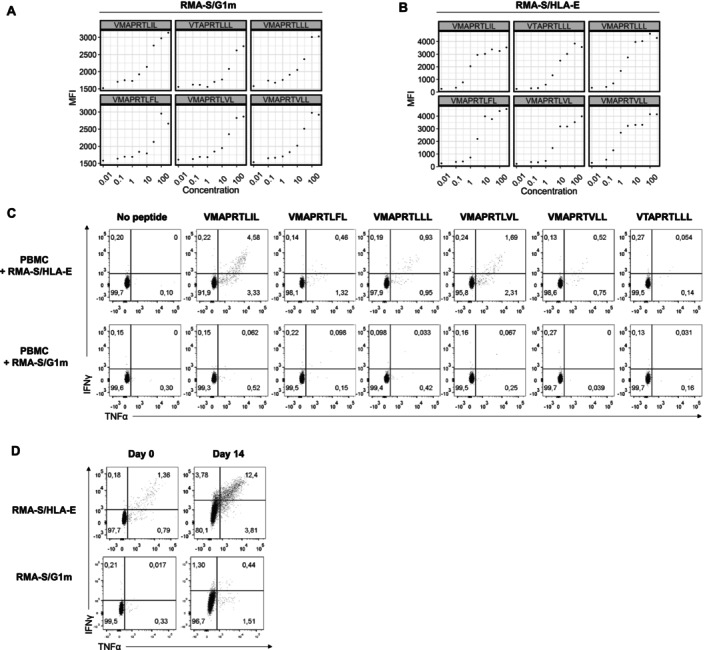
Cytokine production by CD8+ T cells in response to HLA‐I leader sequence peptides presented by RMA‐S/HLA‐E or RMA‐S/G1m. (A, B) Peptide binding assays to RMA‐S/G1m and RMA‐S/HLA‐E were performed as described in Figure 2, testing HLA‐I‐derived leader sequence nonamers at different concentrations (0.01–300 μM); staining of HLA‐G and HLA‐E was respectively carried out with G233 and 3D12 mAbs. (C) PBMC from a donor selected for bearing HLA‐E restricted T cells specific for the VMAPRTLIL peptide were incubated for 6 h with RMA‐S/HLA‐E or RMA‐S/G1m pre‐loaded with different HLA‐I leader signal peptides (100 μM) and washed. Assays were carried out in the presence of anti‐ILT2 mAb (HP‐F1). (D) PBMC were co‐cultured with irradiated RMA‐S/G1m or RMA‐S/HLA‐E for 14 days in the presence of 100 μM VMAPRTLIL, IL‐2 and anti‐ILT2 mAb (HP‐F1). Experiments shown are representative of two performed with two different donors.

On that basis, binding to RMA‐S/G1m cells of the different HLA‐I‐derived leader signal nonamers at different concentrations (0.01–300 μM) was assessed, including RI9L and GR9F as positive and negative controls, respectively. In these assays, HLA‐G was efficiently stabilised by peptides at 3–10 μM (Figure 7A); yet their interaction was weaker than that established with HLA‐E, detectable at 0.3–1 μM. (Figure 7B). In that case, in vitro observations coincided with NetMHCpan4.1 comparison of binding scores for HLA‐G and HLA‐E (Table 2).

Specific alloreactive T‐cell responses to signal peptides presented by HLA‐E are detectable in some individuals, being attributable to infection by a HCMV strain displaying a UL40‐derived peptide different from host HLA‐I signal sequences [10]. This provided an opportunity to address whether HLA‐G might also promote a response to the same peptide antigen. PBMCs from two selected donors bearing T cells specific for HLA‐E/VMAPRTLIL were stimulated either with RMA‐S/HLA‐E or RMA‐S/G1m cells loaded with the different HLA‐I leader peptides, assessing cytokine production. In short‐term (6 h) assays, a specific T‐cell response to VMAPRTLIL was detected only when presented by HLA‐E (Figure 7C).

As previously described for viral peptides, PBMC were co‐cultured for 14 days with VMAPRTLIL‐loaded RMA‐S/HLA‐E or RMA‐S/G1m, subsequently assessing their response in cytokine production assays. An expansion of cells responding to VMAPRTLIL presented by HLA‐E but not HLA‐G was observed (Figure 7D), indicating that the UL40 nonamer was only immunogenic when displayed by the former. Importantly, these observations revealed a clear difference between the ability of both HLA‐Ib molecules for priming a specific T‐cell response to the same antigen, in line with the lack of evidence for an involvement of HLA‐G in the response to other immunogenic viral peptides, described above.

### 3.7 Analysis of the Influence on NK Cell Function of HCMV Peptides Presented by HLA‐E or HLA‐G

**FIGURE 8:**
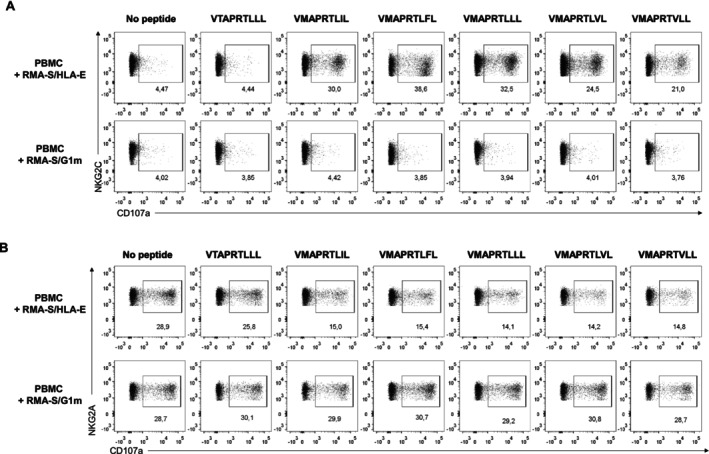
NKG2C+ and NKG2A+ NK cell responses to HLA‐I‐derived peptides presented by RMA‐S/HLA‐E or RMA‐S/G1m cells. (A) PBMC from a selected donor displaying an expansion of the adaptive NKG2C+ NK cell subset were tested in degranulation assays (4 h) against RMA‐S/HLA‐E or RMA‐S/G1m loaded o/n at 26°C with HLA‐I‐derived signal peptides (100 μM). These were washed before co‐culture, carried out in the presence of anti‐ILT2 blocking mAb (HP‐F1). Data are representative of four experiments. (B) Expanded NK cells from a donor displaying NKG2A+ NK cells were tested in degranulation assays against RMA‐S transfectants, as described in (A). Data are representative of three experiments.

It is well established that HLA‐E bound to leader sequence‐derived peptides with Met in P2 constitutes the specific ligand for inhibitory CD94/NKG2A and activating CD94/NKG2C receptors. Their interaction involves HLA‐E α1‐α2 domains, and the affinity is known to be modulated depending on the peptide sequence [48, 59, 60]. Recently, CD94/NKG2A was proposed to interact as well with HLA‐G [61], an issue early considered but ruled out when HLA‐E was proved to be its ligand [5, 6, 39, 62]. Nevertheless, assays were set up to re‐assess whether HLA‐G bound to leader signal/UL40 peptides might be recognised by CD94/NKG2 receptors. In agreement with previous reports, RMA‐S/HLA‐E cells displaying leader sequence peptides with Met in P2 activated degranulation of CD94/NKG2C+ NK cells; by contrast, no response was detected when peptides were presented by HLA‐G (Figure 8A). The putative recognition by CD94/NKG2+ NK cells of HCMV peptides identified to bind both HLA‐G and ‐E was also analysed in HCMV+ donors showing adaptive NKG2C+ NK cell expansions. None of the identified viral peptides loaded either onto RMA‐S/HLA‐E or RMA‐S/G1m cells triggered CD94/NKG2C+ NK cell degranulation (Figure S8).

To circumvent the limited NK cell response to RMA‐S cells, in vitro‐expanded NK cells were employed to check whether CD94/NKG2A might interact with HLA‐G depending on the sequence of bound peptides, inhibiting NK cell activation; degranulation against RMA‐S/HLA‐E loaded with canonical signal peptides was used as control. As expected, inhibition was detected when HLA‐E was loaded with any signal peptide containing Met at P2 (VMA). By contrast, HLA‐G loaded either with HLA‐I/UL40 signal peptides (Figure 8B) or with the identified viral antigens (not shown) was unable to promote any inhibition through NKG2A.

## 4 Discussion

In the present study, we explored the potential role of HLA‐G1 in HCMV antigen presentation by infected cells. To this end, we used an ad hoc experimental setting based on: (i) a melanoma cell line (MSR3‐mel) with an epigenetic silencing of HLA‐I expression to minimise presentation by classical molecules; (ii) an HLA‐G construct (G1m), mutated to prevent binding of its leader peptide to HLA‐E; (iii) an HCMV BACmid (AB8) lacking part of the immune evasion genes that target HLA‐I expression (i.e., US2, US3, US6). This enabled us to identify by MS 22 viral sequences including 9‐mers (*n* = 16), 10‐mers (*n* = 4) and 11‐mers (*n* = 2); importantly 10 viral peptides displayed in both P3 and PΩ anchor residues coincident with those reported for human sequences binding to HLA‐G. HCMV peptides derived from 16 different molecules, six of which were previously reported as antigenic by the assessment of CD4 and CD8 T cell responses to synthetic 15‐mers libraries [49]; two of them correspond to recently identified immunogenic T‐cell epitopes presented by HLA‐C*07:01 and ‐B*07:02 [37].

Most HCMV peptides (*n* = 17) bound in vitro to HLA‐G1 expressed in TAP‐deficient RMA‐S/G1m cells, though their interaction at different concentrations indirectly pointed to affinity differences, and some discordances with in silico predictions by NetMHCpan4.1 were noticed. Of note, HCMV peptides binding to HLA‐G were detected despite that AB8 US10 may downregulate its expression. As US10 has been reported to retain in the ER b2m‐assembled HLA‐G [36], viral peptides in these complexes might have been co‐purified from infected cell lysates and detected by MS; whether other viral sequences would be identified by deleting US10 is uncertain. On the other hand, the possibility that additional HCMV peptides binding to HLA‐G may be identified under different experimental conditions is conceivable. In this regard, HCMV has been reported to generate multiple translation products [54, 63] and, recently, a search using all HCMV ORFs found in Stern‐Ginossar et al. [54] and Erhard et al. [63] identified three additional HCMV sequences predicted to bind HLA‐G (NA: KVFSKPLIL; ORFL62W: RIVETRMTL; UL22A: TTPSKNVTL), which may deserve future attention.

Detection of a set of viral peptides (*n* = 5) which did not bind to RMA‐S/G1m cells was consistent with the absence of conserved anchor residues in P3 and/or P9, suggesting that they might be presented by residual endogenous HLA‐I molecules, co‐purified by the W6/32 mAb. Indeed, NetMHCpan4.1 analysis predicted binding of two of them to MSR3 HLA‐I molecules yet leaving undefined the origin of the other three.

On the other hand, coincident interactions of some HLA‐G‐bound viral peptides with MSR3 HLA‐C, ‐B allotypes and, particularly, with HLA‐E were also predicted. Binding to HLA‐E was confirmed in vitro with RMA‐S/HLA‐E cells in six cases and, reciprocally, NetMHCpan4.1 anticipated an interaction of HLA‐E‐binding nonamers with HLA‐G1. This was verified in vitro with RMA‐S/G1m cells and is consistent with structural similarities reported for HLA‐E and HLA‐G clefts [64]. Of note, one of these sequences (VMAPRTLLL) was identified in the HLA‐I‐bound peptidome from HCMV‐infected MSR3 G1m cells but not from control cultures. However, its viral origin could not be ascertained as MS resolution did not allow to discriminate the difference in P8 between AB8 UL40 (VMAPRTLIL) and MSR3 HLA‐I‐derived peptides (VMAPRTLLL/LIL), as the isobaric residues Leu and Ile cannot be distinguished.

Altogether these results supported the ability of HLA‐G1 to present peptides processed from different HCMV antigens in infected cells, yet did not prove their immunogenicity in the course of natural infection. To address this issue, cytokine production by CD8+ T cells from healthy HCMV(+) donors in response to the viral sequences was assessed in the presence of irradiated RMA‐S/G1m or RMA‐S/b2m cells and a mAb to block the inhibitory ILT2 receptor. No cytokine production was observed in response to any of the identified peptides in short‐term (6 h) assays. However, after stimulation in the presence of IL‐2 for 14 days, CD8+ T cells responding to individual viral peptides were detected in some individuals, as reported by Lübke et al. [37]. Of note, activation was observed regardless of the presence of RMA‐S/G1m cells indicating that viral antigens might be presented either by donor HLA‐I molecules, as predicted in some cases by NetMHCpan4.1 in silico analysis, and/or by HLA‐G expressed in PBMC, an issue which remains controversial. In line with previous reports [65, 66], a limited HLA‐G1 transcription was detected by RT‐PCR in some PBMC samples, and a minor T‐cell subset was stained by three different mAbs reported to react with HLA‐G [17] (unpublished results). However, T‐cell activation in response to HCMV peptides was not inhibited by anti HLA‐G mAbs (i.e., G233, 87G) and, importantly, was undetectable when T cells were re‐challenged with peptides loaded onto RMA‐S/G1m cells, instead of in soluble form.

These observations indicated that some viral peptides binding to HLA‐G in infected cells may be immunogenic when presented by other HLA‐I molecules but not by HLA‐G. A caveat was the lack of a positive control in the experiments, given the unprecedented identification of T cells specific for HLA‐G‐restricted antigens. As a surrogate approach, we considered that some individuals bear circulating T cells specific for HLA‐E bound to a canonical HLA‐I/UL40 nonamer (VMAPRTLIL), shown to interact also with HLA‐G. PBMC from two donors with this profile were stimulated with the peptide loaded onto RMA‐S/G1m or RMA‐S/HLA‐E cells. CD8+ T‐cell cytokine production was exclusively detected in response to the latter, indirectly indicating a lower or lack of immunogenicity of the same epitope presented by HLA‐G.

Several factors may underlie an inefficient HLA‐G1‐restricted T cell priming by HCMV antigens. First, its limited systemic expression and restricted distribution relative to other HLA‐I molecules [67, 68] likely minimises HLA‐G1 participation in the TCR repertoire development and antigen presentation processes. Similarly, the relatively reduced size of the HLA‐C peptidome compared to that of HLA‐A and ‐B molecules may be related with its lower expression levels [37]. Second, HLA‐G is considered to play a tolerogenic function by different mechanisms [18, 69], particularly interacting with ILT2 and ILT4 inhibitory receptors with higher affinity than other HLA‐I molecules [70]. Yet, T‐cell unresponsiveness upon re‐stimulation with HLA‐G‐bound viral peptides was not reverted by a blocking anti ILT2 mAb. Third, HLA‐G molecules are assembled as homodimers, in which disulphide‐linked monomers are predicted to adopt a head to tail conformation [28, 70], which might be unfavourable for TCR interaction and CD8+ T‐cell priming; in that case, peptides might only contribute to stabilise HLA‐G surface expression. Finally, the possibility that soluble HLA‐G presenting HCMV peptides might competitively inhibit specific T‐cell activation appears unlikely, considering the reduced number of TCR‐MHC interactions required to trigger a T‐cell response.

Together with HLA‐E and ‐C molecules, HLA‐G is displayed by embryonic extravillous cytotrophoblasts (EVT) which interact at early stages of pregnancy with maternal decidual NK (dNK) cells, T lymphocytes and myelomonocytic cells [71]. dNK cells express KIRs and CD94/NKG2A inhibitory receptors respectively specific for HLA‐C and HLA‐E [71]. Balanced interactions of KIRs with embryonic/foetal HLA‐C allotypes are important for normal placentation (e.g., spiral artery remodelling) and specific combinations of maternal KIR and paternal HLA‐I genotypes have been associated with the incidence of gestational complications (e.g., preeclampsia) [72, 73]. HLA‐G has been proposed to promote tolerance and vascular remodelling at the maternal–embryonic interface through different mechanisms [19, 71, 74], including direct engagement of leukocyte receptors (i.e., ILT2, ILT4) and enhancement of the CD94/NKG2 affinity for HLA‐E by loading the latter with the HLA‐G unique leader sequence (VMAPRTLFL) [48, 60]. Whether HLA‐G‐bound viral peptides may influence its proposed interaction with KIR2DL4 in an endosomal compartment [75, 76, 77] is uncertain.

Besides that, the tolerogenic milieu at the maternal‐foetal interface may favour vertical transmission of pathogens causing congenital infections. Yet, the incidence of viral infections (e.g., HCMV) at early stages of gestation has been associated to miscarriage, presumably as a result of the maternal immune response promoting inflammation and dNK cell activation [78, 79]. Whether some HLA‐G‐binding HCMV peptides might maintain its expression in infected cytotrophoblasts, enabling inhibitory ILT2 and ILT4 engagement, is uncertain. On the other hand, UL40‐derived peptides replicating endogenous HLA‐I leader sequences may bind to HLA‐E, being recognised with different affinities by CD94/NKG2 receptors [80]. Though their role in HLA‐G recognition was early dismissed [62], an interaction with CD94/NKG2A has been recently proposed [61], yet its putative functional relevance is not supported by our data. In fact, HLA‐G bound to HLA‐I leader sequence/UL40‐derived nonamers and viral peptides failed to modulate NK cell functions through either NKG2A or NKG2C.

Antigen presentation by HLA‐A and ‐B allotypes appears to dominate the specific CD8+ T cell response to HCMV antigens, but HLA‐C‐restricted T cells may also contribute to antiviral defence [81, 82, 83]. In this study, several HLA‐G‐binding viral peptides were predicted in silico to potentially interact as well with HLA‐C allotypes and one of them (UL78 (KR9L)) was previously reported to activate T cells [37], coinciding with our results. Their response might be relevant in case of HCMV reactivation/reinfection affecting EVT and, moreover, could promote a potential cross‐reactivity with paternal HLA‐C alloantigens [78, 79, 84]. According to our results, a similar role for HLA‐G appears unlikely, though the possibility that priming by viral antigens might occur in the inflammatory context associated with HCMV infection of HLA‐G+ EVT is not ruled out.

Given their limited polymorphism, HLA class Ib molecules presenting tumour antigens have been considered potential targets for specific T cell‐mediated cancer immunotherapy [85]. Consistent with its broad peptidome [30], HLA‐G may also display neo‐ and minor histocompatibility allo‐antigens. Yet, according to our observations, the lack of immunogenicity of HLA‐G‐presented viral antigens raises a concern for its practical implication in this scenario.

## Author Contributions

Mireia Altadill performed experiments, analysed and interpreted data and contributed to manuscript writing. Gemma Heredia performed experiments. Iñaki Álvarez obtained peptide preparations for MS analysis and contributed to peptidome analysis and interpretation. Michelle Ataya, Elisenda Alari‐Pahissa and Aura Muntasell contributed to develop the technical setup for assessing the T and NK cell response to viral peptides. Manuel Llano participated in the generation of HLA‐G transfectants for functional studies. Carlos Vilches provided information on donor HLA typing and designed RT‐PCR analysis. Jonas Fuchs performed AB8 sequencing assembly and analysis. Hartmut Hengel and Anne Halenius furnished key advice, materials and technical support for setting up HCMV infection conditions and contributed to virus sequence assembly and analysis. All co‐authors revised the manuscript. Miguel López‐Botet conceived and designed the study, directed research and wrote the manuscript integrating the intellectual input and revision of all co‐authors.

## Conflicts of Interest

The authors declare no conflicts of interest.

## Supporting information


**Figure S1.** Schematic genome organization of HCMV BAC clone AB8.

**Figure S2.** Phenotype of MSR3 G1m (Mock and HCMV‐AB8‐infected).

**Table S3.** Detailed features of HCMV peptides identified from infected MSR3 G1m cells.

**Table S4.** In silico prediction (NetMHCpan4.1) of HCMV peptide interactions with endogenous HLA‐I fromMSR3 G1m cells.

**Figure S5.** Specific cytokine production by CD8+ T cells in response to HCMV peptides.

**Table S6.** Summarized information on the identified HCMV peptides.

**Table S7.** In silico predictions (NetMHCpan4.1) of HCMV peptides binding to HLA‐I of a blood donor.

**Figure S8.** NK cells response to HCMV peptides presented by RMA‐S/HLA‐E or RMA‐S/G1m cells.

Data S1.

## Data Availability

The data that support the findings of this study are available from the corresponding author upon reasonable request.
