# Supplementary material for: Human Cytomegalovirus Antigen Presentation by HLA‐G in Infected Cells
Source: HLA. 2025 May 10;105(5):e70089. doi: 10.1111/tan.70089 (PMC12065092; doi:10.1111/tan.70089)
Supplement: Supplementary file 1 — Figure S1. Schematic genome organization of HCMV BAC clone AB8. [file TAN-105-e70089-s001.pdf]

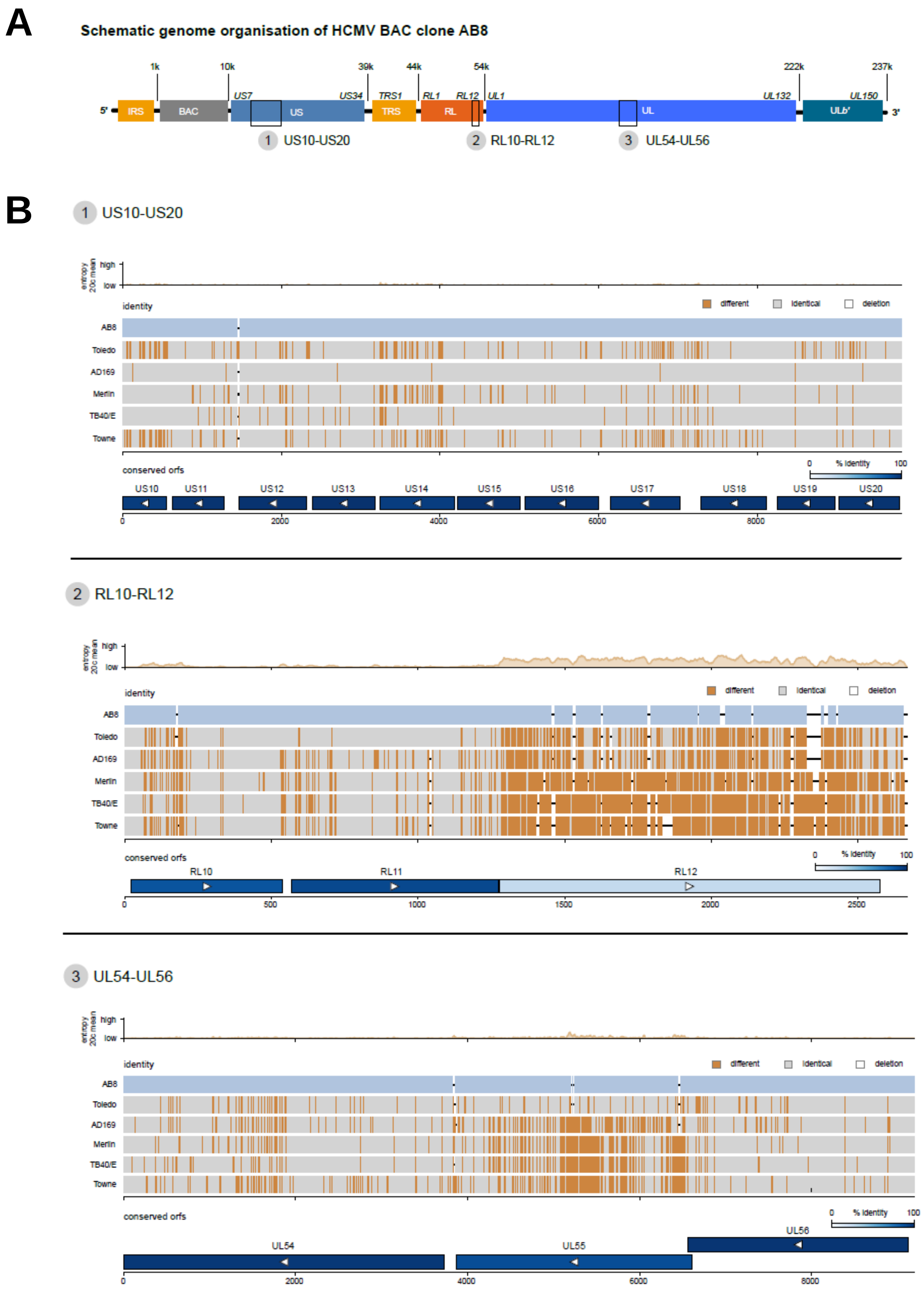
A
Schematic genome organisation of HCMV BAC clone AB8
1k
10k
39k
44k
54k
222k
237k
5'
IRS
BAC
US7
US
US34
TRS1
TRS
RL1
RL
RL12
UL1
UL
UL132
UL150
ULb'
3'
1 US10-US20
2 RL10-RL12
3 UL54-UL56
B
1 US10-US20
entropy 20c mean
high
low
identity
different
identical
deletion
AB8
Toledo
AD169
Merlin
TB40/E
Towne
conserved orfs
0
% identity
100
US10
US11
US12
US13
US14
US15
US16
US17
US18
US19
US20
0
2000
4000
6000
8000
2 RL10-RL12
RL10
RL11
RL12
0
500
1000
1500
2000
2500
3 UL54-UL56
UL54
UL55
UL56
0
2000
4000
6000
8000

**S1 Fig. Schematic genome organization of HCMV BAC clone AB8. (A)** Schematic representation of the genome organisation of HCMV BAC clone AB8. IRS – internal repeat short, BAC – bacterial artificial chromosome, TRS – terminal repeat short, RL – repeat long, US - unique short, UL - unique long. (**B)** Alignments of selected regions of AB8 and the HCMV strains Towne (GQ121041.1), Toledo (KY002201.1), AD169 (FJ527563.1), TB40/E (KF297339), and Merlin (OK000909). Each plot consist of three panels. Top: 40 nucleotide rolling average Shannon's normalized entropy. Middle: Schematic alignment overview displaying differences to AB8. Bottom: Conserved open reading frames (ORFs) defined as ORFs that have a conserved start and stop codon in every sequence of the alignment and no additional stop codons within the respective frames. The coloration or the ORF annotation corresponds to the mean identity over the respective alignment regions.
