# Supplementary material for: Human Cytomegalovirus Antigen Presentation by HLA‐G in Infected Cells
Source: HLA. 2025 May 10;105(5):e70089. doi: 10.1111/tan.70089 (PMC12065092; doi:10.1111/tan.70089)
Supplement: Supplementary file 2 — Figure S2. Phenotype of MSR3 G1m (Mock and HCMV‐AB8‐infected). [file TAN-105-e70089-s004.pdf]

A

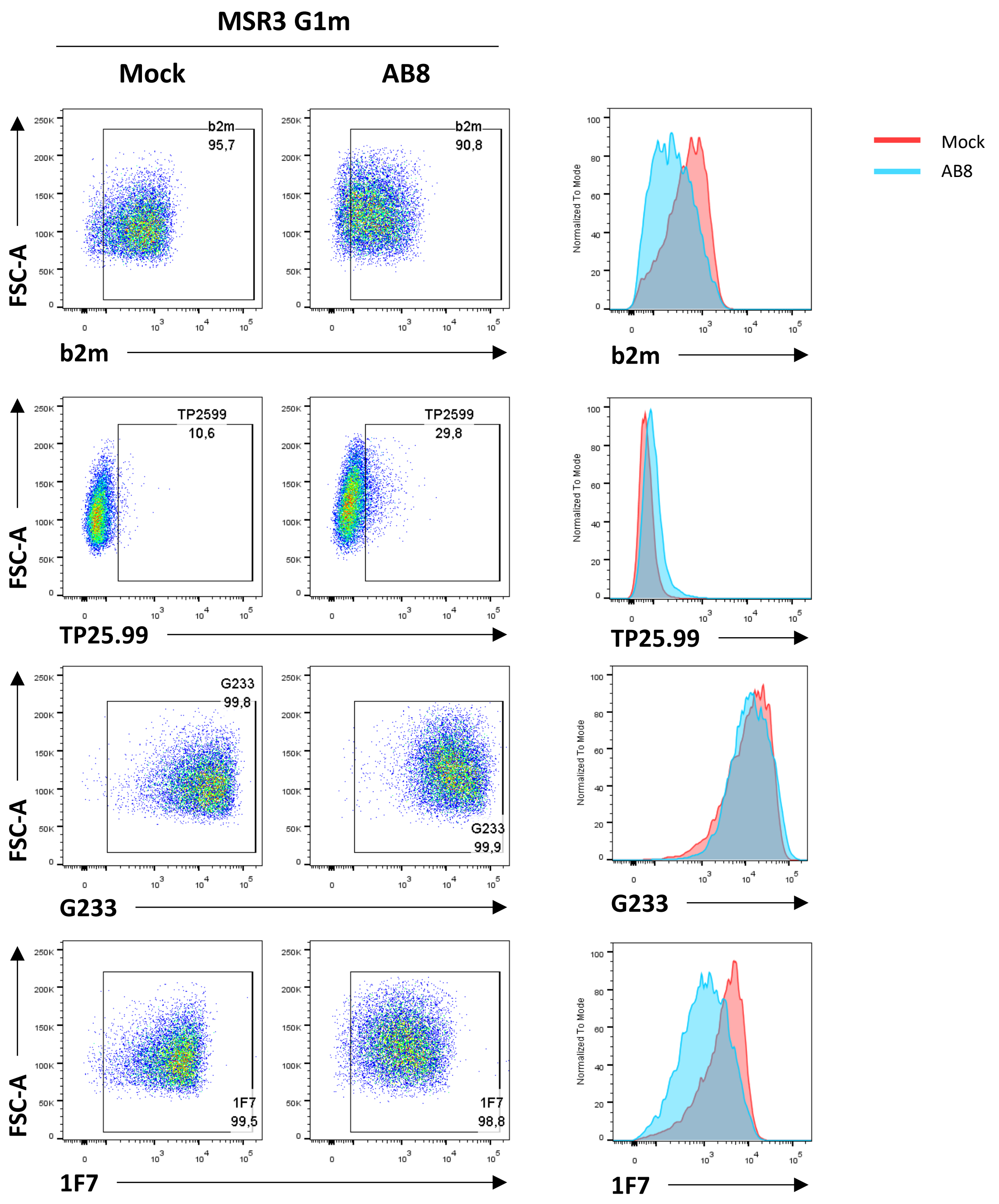
MSR3 G1m
Mock
AB8
b2m
95,7
90,8
TP2599
10,6
29,8
G233
99,8
99,9
1F7
99,5
98,8
FSC-A
Normalized To Mode
b2m
TP25.99
G233
1F7
Mock
AB8

B

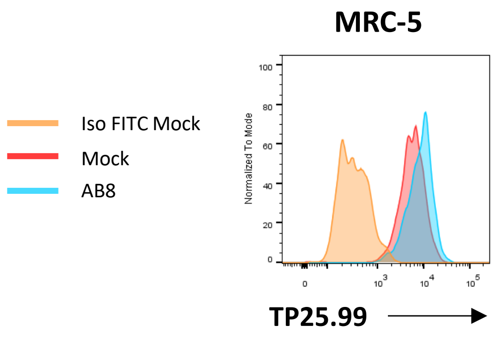
MRC-5
Iso FITC Mock
Mock
AB8
Normalized To Mode
TP25.99

S2 Fig. Phenotype of MSR3 G1m (Mock and HCMV-AB8-infected). (A) Flow cytometry staining of mock and HCMV-infected MSR3 G1m cells by direct IF with anti-b2m, TP25.99SF (anti HLA-A,B,C, E), HP-1F7 (anti HLA-I mAbs) and by indirect IF with G233 (anti HLA-G) mAb. (**B)** Control TP25.99SF staining of mock and HCMV-infected MRC-5.
