# Supplementary material for: Human Cytomegalovirus Antigen Presentation by HLA‐G in Infected Cells
Source: HLA. 2025 May 10;105(5):e70089. doi: 10.1111/tan.70089 (PMC12065092; doi:10.1111/tan.70089)
Supplement: Supplementary file 4 — Table S4. In silico prediction (NetMHCpan4.1) of HCMV peptide interactions with endogenous HLA‐I fromMSR3 G1m cells. [file TAN-105-e70089-s003.pdf]

S4 Table. ***In silico*** **prediction (NetMHCpan4.1) of HCMV peptide interactions with endogenous HLA-I from MSR3 G1m cells.**

		Peptide	A*01:01	A*11:01	B*37:01	B*52:01	C*06:02	C*12:02	G*01:01	Best rank	*In vitro* binding to RMA-S/G1m
s.b.	SM9L	SMPELSLTL	5.2572	9.0260	**1.1054**	**0.3335**	**0.4622**	**0.6971**	**0.01**	**G*01:01**	***
	VQ9L	VQPRQTVEL	8.7612	16.3710	**0.4452**	**0.4619**	**0.8705**	**1.0008**	**0.0487**	**G*01:01**	**
	VS9L	VSPGKEVTL	6.4012	19.8710	2.8986	**1.8890**	**1.0364**	**1.1940**	**0.0491**	**G*01:01**	**
	RF11L	RFPERAGYEKL	28.4333	17.5800	3.3055	19.4387	7.6250	31.7500	**0.0715**	**G*01:01**	***
	HQ9L	HQPRGRILL	8.7863	18.0263	**1.1222**	**1.6423**	**1.1920**	2.6074	**0.0717**	**G*01:01**	***
	KI9F	KIPLRRVIF	17.9720	15.2987	10.1704	11.8489	5.1482	4.3271	**0.0971**	**G*01:01**	**
	RE9F	REPPHRALF	3.1687	12.8820	**0.0501**	**1.9091**	**1.4598**	3.3178	**0.1598**	B*37:01	
	RL9L	RLAPYPADL	10.9747	14.7745	3.3644	3.6257	2.1514	**1.6857**	**0.3077**	**G*01:01**	**
w.b.	QI9F	QIVPRGVMF	5.4486	8.5067	5.9228	4.0634	3.8443	**0.6656**	**0.52**	**G*01:01**	**
	VRP9L	VRPTRQLVL	15.5570	22.7778	3.3076	5.5626	**0.0288**	4.6948	**0.5699**	C*06:02	***
	HL9L	HLVPSGNVL	8.0000	22.1111	3.6899	2.0127	2.5729	**0.5377**	**0.9338**	C*12:02	***
	VM9L	VMVSSSLVL	17.6822	18.1842	6.4983	**1.0406**	5.1171	**1.4349**	**1.3497**	B*52:01	**
	RT10L	RTGSLHHFEL	10.8023	17.0200	16.1196	25.7421	14.6261	10.8143	**1.598**	**G*01:01**	**
n.b.	KR9L	KRAMYSVEL	20.6338	20.0000	2.2152	6.0752	**0.0553**	4.3388	2.5473	C*06:02	***
	VRS9L	VRSRDSLLL	13.3394	25.2000	4.8038	8.1173	**0.2022**	9.6300	2.8248	C*06:02	**
	SG10L	SGVRRPFTEL	27.5429	17.6200	17.2260	11.6434	13.8298	7.0216	3.7952	unk.	**
	RI11L	RIVEPLESGRL	25.2683	7.5721	3.0324	13.4838	29.5455	11.0222	4.0386	unk.	**
	SP10L	SPSRDRFVQL	12.3764	15.1039	5.6032	8.9754	5.2376	3.9371	4.3484	unk.	
	RP9L	RPRLTLHDL	19.4444	23.2667	4.2204	5.5287	9.8493	7.5300	5.3686	unk.	
	TL10L	TLKGLRKLIL	30.3929	53.7500	30.8621	21.9101	16.9512	24.1034	9.3978	unk.	**
	SE9V	SETTVHVVV	13.9495	26.4000	**0.0900**	**0.3229**	4.2109	9.6941	22.1101	B*37:01	
	VR9K	VRLSDLRLK	63.5714	7.1237	39.6000	40.4878	6.9065	28.0588	43.6364	unk.	

***: Strong *in vitro* binding to HLA-G

**: Weak *in vitro* binding to HLA-G

Dark blue: Strong binder according to NetMHCpan4.1 (<0.5% rank)

Light blue: Weak binder according to NetMHCpan4.1 (0.5<x<2% rank)

Uncoloured: No binder according to NetMHCpan4.1 (>2% rank)

s.b.: Strong binder to HLA-G according to NetMHCpan4.1

w.b.: Weak binder to HLA-G according to NetMHCpan4.1

n.b.: No binder to HLA-G according to NetMHCpan4.1
