# Supplementary material for: Human Cytomegalovirus Antigen Presentation by HLA‐G in Infected Cells
Source: HLA. 2025 May 10;105(5):e70089. doi: 10.1111/tan.70089 (PMC12065092; doi:10.1111/tan.70089)
Supplement: Supplementary file 5 — Figure S5. Specific cytokine production by CD8+ T cells in response to HCMV peptides. [file TAN-105-e70089-s008.pdf]

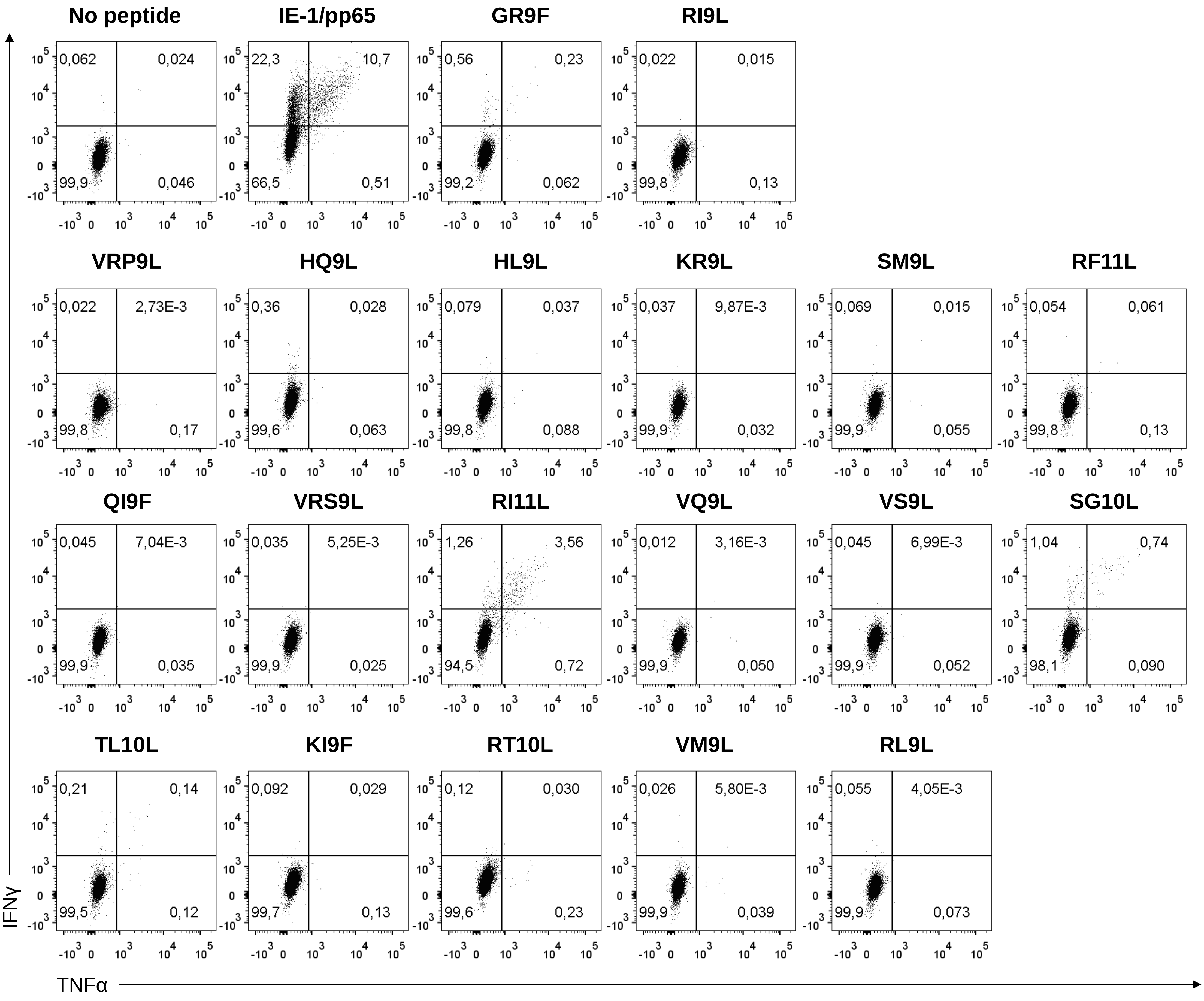

S5 Fig. Specific cytokine production by CD8+ T cells in response to HCMV peptides. PBMC were cultured for 14 days in the presence of individual peptides (100 µM) and IL-2 (200 UI/mL) added at different time points (see Methods). Subsequently, cells were re-stimulated for 6 h with the corresponding peptide (100 µM) and cytokine production (TNFα, IFNγ) was analyzed by flow cytometry. IE-1/pp65 peptides were used as a positive control of specific-T cell expansion. Data representative of 15 assays correspond to an individual donor
