# Supplementary material for: Human Cytomegalovirus Antigen Presentation by HLA‐G in Infected Cells
Source: HLA. 2025 May 10;105(5):e70089. doi: 10.1111/tan.70089 (PMC12065092; doi:10.1111/tan.70089)
Supplement: Supplementary file 7 — Table S7. In silico predictions (NetMHCpan4.1) of HCMV peptides binding to HLA‐I of a blood donor. [file TAN-105-e70089-s009.pdf]

S7 Table. In silico predictions (NetMHCpan4.1) of HCMV peptides binding to HLA-I of a blood donor.

Peptide	A*02:01	A*03:01	B*07:02	B*44:03	C*02:02	C*07:02	G*01:01	E*01:01*
SMPELSLTL	**0.1093**	8.3738	2.6165	8.2906	**0.7831**	**0.2102**	**0.01**	**0.0052**
VQPRQTVEL	2.3640	20.1087	5.8860	7.3605	**1.4187**	**0.6008**	**0.0487**	**0.0084**
VSPGKEVTL	5.4266	24.4800	3.9647	17.6585	**1.5750**	**1.2763**	**0.0491**	**0.0178**
RFPERAGYEKL	21.4605	17.9630	6.5905	5.5781	30.8000	4.4112	**0.0715**	**1.451**
HQPRGRILL	7.6027	18.5857	3.8915	7.1988	2.9347	**1.1918**	**0.0717**	**0.0305**
KIPLRRVIF	25.4146	11.4213	2.1541	22.9412	3.2865	2.8622	**0.0971**	**0.2041**
REPPHRALF	20.1034	14.1329	3.5709	**0.3165**	3.6349	**1.0572**	**0.1598**	**0.1751**
RLAPYPADL	**0.2182**	5.2122	2.4280	11.6994	2.2561	**1.4833**	**0.3077**	**0.1473**
QIVPRGVMF	10.2718	7.8760	2.2539	5.7894	**0.6091**	2.0628	**0.52**	**0.8935**
VRPTRQLVL	17.2323	21.1026	7.4868	14.7973	7.1291	**0.0251**	**0.5699**	**0.1899**
HLVPSGNVL	**1.4550**	13.9409	**1.1726**	9.5745	**1.0399**	**1.4438**	**0.9338**	**0.6969**
VMVSSSLVL	**1.5160**	11.3120	3.8244	17.9756	2.6970	2.6054	**1.3497**	**0.0811**
RTGSLHHFEL	14.5822	18.6143	6.8985	37.6667	8.5076	15.5273	**1.598**	2.3743
KRAMYSVEL	12.9226	14.1899	6.3525	9.6064	6.5032	**0.1196**	2.5473	**1.0987**
VRSRDSLLL	18.8790	25.0952	15.1778	12.4000	9.8522	**0.4413**	2.8248	2.3505
SGVRRPFTEL	18.1855	14.5760	**0.8436**	39.0000	6.7334	10.6851	3.7952	2.8168
RIVEPLESGRL	**1.4974**	4.1116	4.4337	13.0526	7.8413	26.3000	4.0386	4.9499
SPSRDRFVQL	17.9355	15.0667	**0.0296**	11.8834	5.0151	3.7032	4.3484	**1.6699**
RPRLTLHDL	24.8750	22.2571	**0.0052**	15.3387	13.9831	6.0037	5.3686	3.9650
TLKGLRKLIL	9.4528	27.0625	8.1103	38.0000	22.0667	19.3462	9.3978	7.3850
SETTVHVVV	15.5556	28.5333	9.1022	**0.4498**	12.6853	5.8845	22.1101	3.6696
VRLSDLRLK	38.1000	6.4584	57.7778	12.8240	30.6000	4.4627	43.6364	29.5000

*Prediction for E*01:01 is also valid for E*01:03

Dark blue: strong binder according to NetMHCpan4.1 (<0.5% rank)

Light blue: weak binder according to NetMHCpan4.1 (0.5<x<2% rank)
