# Supplementary material for: Human Cytomegalovirus Antigen Presentation by HLA‐G in Infected Cells
Source: HLA. 2025 May 10;105(5):e70089. doi: 10.1111/tan.70089 (PMC12065092; doi:10.1111/tan.70089)
Supplement: Supplementary file 8 — Figure S8. NK cells response to HCMV peptides presented by RMA‐S/HLA‐E or RMA‐S/G1m cells. [file TAN-105-e70089-s006.pdf]

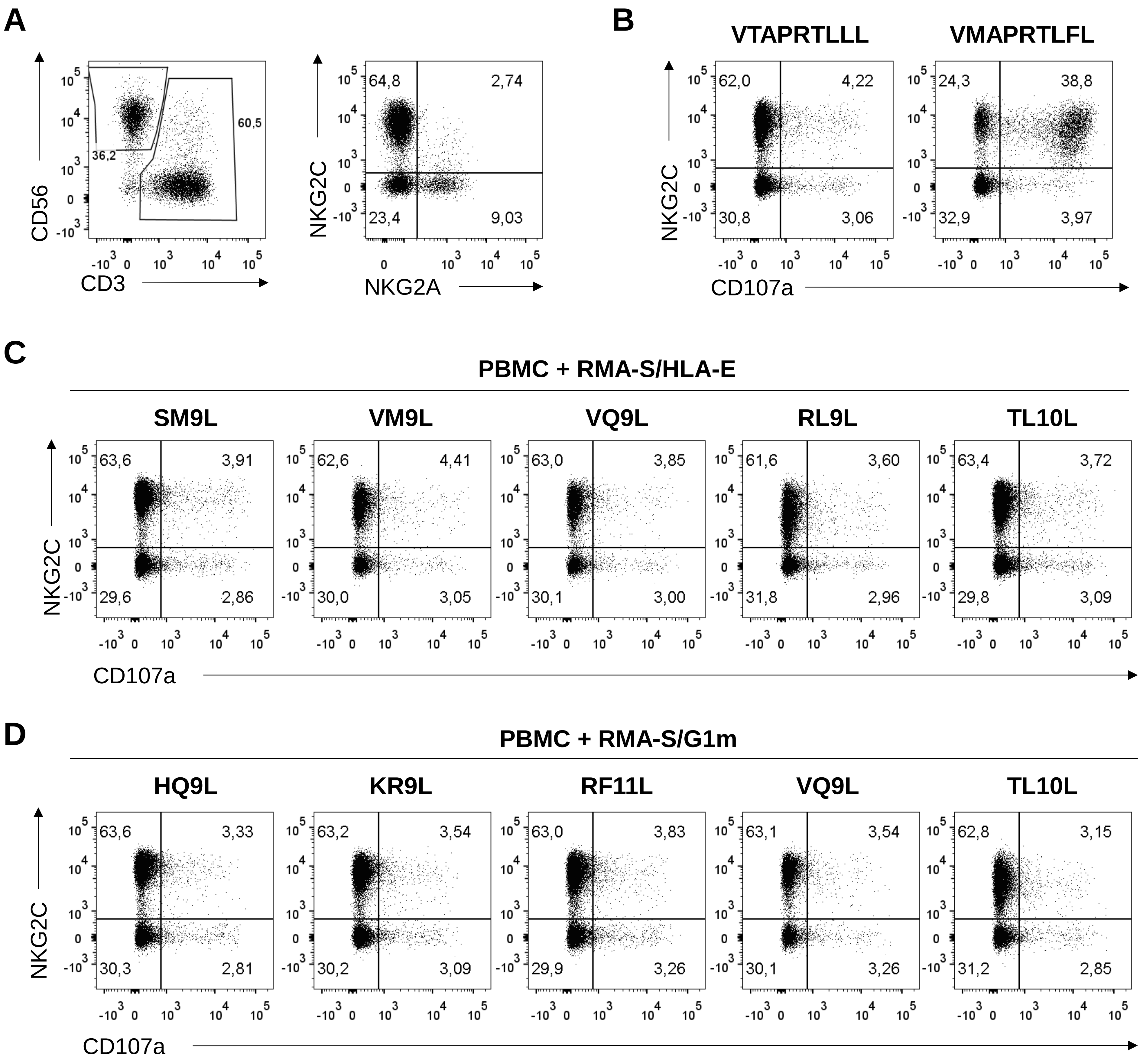

S8 Fig. NK cells response to HCMV peptides presented by RMA-S/HLA-E or RMA-S/G1m cells. (A-B) NK cells from a selected donor displaying an expansion of the adaptive NKG2C+ subset were tested in degranulation assays against RMA-S/HLA-E cells loaded with VTAPRTLLL and VMAPRTLFL HLA-I/UL40 nonamers, as negative and positive controls, respectively. **(C-D)** NK cell degranulation against RMA-S/HLA-E (C) or RMA-S/G1m (D) loaded with HCMV peptides.
