# Supplementary material for: Human Cytomegalovirus Antigen Presentation by HLA‐G in Infected Cells
Source: HLA. 2025 May 10;105(5):e70089. doi: 10.1111/tan.70089 (PMC12065092; doi:10.1111/tan.70089)
Supplement: Supplementary file 9 — Data S1. [file TAN-105-e70089-s005.docx]

**Supplementary Materials and Methods**

**AB8 HCMV characterization and stock production**

AB8 sequencing was carried out as follows. Viral supernatant (300 µL) was sterile filtered with 0.45 µm PES syringe filters. DNA was purified with the Monarch Genomic DNA Purification Kit (New England Biolabs, Frankfurt am Main, Germany). Paired end Illumina sequencing libraries were prepared using the NEBNext Ultra II FS DNA Library Prep Kit (E6177, NEB, Frankfurt am Main, Germany). Normalized and pooled libraries were denatured with 0.2 N NaOH and sequenced on an Illumina MiSeq instrument using the 300-cycle MiSeq Reagent Kit v2 (MS-102-2002, Illumina). To reconstruct the viral genome, a de novo assembly pipeline developed on the Galaxy platform (1) was used (available at: https://usegalaxy.eu/u/jonasfuchs/w/viral-de-novo-assembly-pipeline). Briefly, raw reads were quality controlled using fastp v.0.23.2 (2) and pre-filtered for viral reads using Kraken2 v.2.1.1 (3) with prebuilt Viral Refseq indexes. The non-viral reads were mapped to the human genome hg19 using BWA-MEM v.0.7.17 (4). The pre-filtered viral reads and non-human reads were used as the input for the three different de novo assemblers Megahit v.1.2.9 (5), velvet v.1.2.10 (6), and spades v.3.15.4 (7). The contigs of all three assemblers were used to assemble the full-length viral genome with cap3 v.10.2011 (8). The final genome was error corrected by remapping the initial quality controlled reads to the final contig using BWA-MEM. Lastly, a consensus sequence was generated with ivar v.1.4.2 (9) and the genome annotated with Prokka v.1.14.6 (10). The Prokka prediction for the coding sequences was manually corrected. To compare selected regions of AB8 to other HCMV strains, MAFFT v.7.526 (10.1093/nar/gkf436) was utilized. The alignments were visualized with python3.11 using the packages matplotlib v.3.9 and msaexplorer v.0.1 (https://github.com/jonas-fuchs/MSAexplorer).

**REFERENCES**

1. Galaxy platform for accessible, reproducible, and collaborative data analyses: 2024 update. Nucleic Acids Research. 2024;52:W83–94.

2. Chen S, Zhou Y, Chen Y, Gu J. fastp: an ultra-fast all-in-one FASTQ preprocessor. bioRxiv; 2018. p. 274100.

3. Lu J, Rincon N, Wood DE, Breitwieser FP, Pockrandt C, Langmead B, et al. Metagenome analysis using the Kraken software suite. Nat Protoc. 2022 Dec;17(12):2815–39.

4. Li H, Durbin R. Fast and accurate long-read alignment with Burrows–Wheeler transform. Bioinformatics. 2010 Mar 1;26(5):589–95.

5. Li D, Liu CM, Luo R, Sadakane K, Lam TW. MEGAHIT: an ultra-fast single-node solution for large and complex metagenomics assembly via succinct de Bruijn graph. Bioinformatics. 2015 May 15;31(10):1674–6.

6. Zerbino DR, McEwen GK, Margulies EH, Birney E. Pebble and Rock Band: Heuristic Resolution of Repeats and Scaffolding in the Velvet Short-Read de Novo Assembler. PLOS ONE. 2009;4(23):e8407.

7. Nurk S, Bankevich A, Antipov D, Gurevich A, Korobeynikov A, Lapidus A, et al. Assembling Genomes and Mini-metagenomes from Highly Chimeric Reads. In: Deng M, Jiang R, Sun F, Zhang X, editors. Research in Computational Molecular Biology. Berlin, Heidelberg: Springer; 2013. p. 158–70.

8. Huang X, Madan A. CAP3: A DNA Sequence Assembly Program. Genome Res. 1999;9:868–77.

9. Grubaugh ND, Gangavarapu K, Quick J, Matteson NL, De Jesus JG, Main BJ, et al. An amplicon-based sequencing framework for accurately measuring intrahost virus diversity using PrimalSeq and iVar. Genome Biol. 2019 Jan 8;20(1):8.

10. Seemann T. Prokka: rapid prokaryotic genome annotation. Bioinformatics. 2014 Jul 15;30(14):2068–9.
